# Allosteric regulation of Senecavirus A 3C^pro^ proteolytic activity by an endogenous phospholipid

**DOI:** 10.1371/journal.ppat.1011411

**Published:** 2023-05-30

**Authors:** Hai-Fan Zhao, Liang Meng, Zhi Geng, Zeng-Qiang Gao, Yu-Hui Dong, Hai-Wei Wang, Heng Zhang

**Affiliations:** 1 School of Life Sciences, University of Science and Technology of China, Hefei, China; 2 Beijing Synchrotron Radiation Facility, Institute of High Energy Physics, Chinese Academy of Sciences, Beijing, China; 3 State Key Laboratory for Animal Disease Control and Prevention, Harbin Veterinary Research Institute, Chinese Academy of Agricultural Sciences, Harbin, China; University of Maryland, UNITED STATES

## Abstract

Seneca virus A (SVA) is an emerging novel picornavirus that has recently been identified as the causative agent of many cases of porcine vesicular diseases in multiple countries. In addition to cleavage of viral polyprotein, the viral 3C protease (3C^pro^) plays an important role in the regulation of several physiological processes involved in cellular antiviral responses by cleaving critical cellular proteins. Through a combination of crystallography, untargeted lipidomics, and immunoblotting, we identified the association of SVA 3C^pro^ with an endogenous phospholipid molecule, which binds to a unique region neighboring the proteolytic site of SVA 3C^pro^. Our lipid-binding assays showed that SVA 3C^pro^ displayed preferred binding to cardiolipin (CL), followed by phosphoinositol-4-phosphate (PI4P) and sulfatide. Importantly, we found that the proteolytic activity of SVA 3C^pro^ was activated in the presence of the phospholipid, and the enzymatic activity is inhibited when the phospholipid-binding capacity decreased. Interestingly, in the wild-type SVA 3C^pro^-substrate peptide structure, the cleavage residue cannot form a covalent binding to the catalytic cysteine residue to form the acyl-enzyme intermediate observed in several picornaviral 3C^pro^ structures. We observed a decrease in infectivity titers of SVA mutants harboring mutations that impaired the lipid-binding ability of 3C^pro^, indicating a positive regulation of SVA infection capacity mediated by phospholipids. Our findings reveal a mutual regulation between the proteolytic activity and phospholipid-binding capacity in SVA 3C^pro^, suggesting that endogenous phospholipid may function as an allosteric activator that regulate the enzyme’s proteolytic activity during infection.

## Author summary

Seneca virus A (SVA) is a novel picornavirus and there have been several recent outbreaks that caused a great threat to the swine industry in some countries. The protease 3C^pro^ is responsible for the cleavage of viral polyprotein and it can also cleave several host proteins enabling SVA to escape antiviral innate immune responses. We identified an endogenous phospholipid molecule that binds to a unique region in SVA 3C^pro^ by a combination of crystallography, untargeted lipidomics, and immunoblotting. The preferred phospholipid types include cardiolipin, phosphoinositol-4-phosphate and sulfatide. We found the protease activity can be significantly inhibited when the phospholipid-binding capacity decreases. Meanwhile, a decrease in infectivity titers of SVA mutants harboring mutations that impair the lipid-binding ability of 3C^pro^ was observed. Our findings suggest the phospholipid may function as an allosteric activator to regulate SVA 3C^pro^ proteolytic activity associated with the viral replication and infection.

## Introduction

Senecavirus A (SVA) is a non-enveloped positive single-stranded RNA virus that belongs to the family Picornaviridae [1]. SVA infection can cause many vesicular diseases similar to those of foot-and-mouth disease [2–4], and poses a great threat to the swine industry. In recent years, there have been several outbreaks of this emerging picornavirus reported in the United States [5,6], Brazil [4,7,8], and China [3,9]. SVA is of pathogenic importance in the swine industry. Besides, SVA has the potential in oncolytic virotherapy due to its oncolytic potential and ability to penetrate solid tumors through the vascular system [10,11].

The full-length SVA genome is approximately 7.3 kb in length and contains a single open reading frame (ORF) encoding a single polyprotein that undergoes proteolytic processing and cleavage by the viral 3C protease (3C^pro^) into four structural proteins (VP4-VP2-VP3-VP1) and eight non-structural proteins (L-2A-2B-2C-3A-3B-3C-3D) [1] (S1 Table). In addition to its role in viral replication, several recent studies have revealed that SVA 3C^pro^ plays multiple roles in virus-host interactions that enable SVA to escape antiviral innate immune responses. For example, SVA can inhibit antiviral responses by targeting different adaptors involved in type I interferon (IFN) signaling, including mitochondrial antiviral signaling (MAVS), Toll/interleukin 1 (IL-1) receptor domain-containing adaptor inducing IFN-β (TRIF), and TRAF family member-associated NF-κB activator (TANK), through 3C^pro^-mediated cleavage [12]. SVA 3C^pro^ also cleaves a poly(A) binding protein cytoplasmic 1 (PABPC1) to promote viral replication [13], and targets porcine gasdermin D (pGSDMD) for cleavage to induce pyroptosis [14]. SVA 3C^pro^ may also reduce IRF3 and IRF7 protein levels and phosphorylation levels via its protease activity, which cause the reduced transcription of IFN-β, IFN-α1, IFN-α4, and ISG54 genes [15]. SVA 3C^pro^ was also found to act as a viral deubiquitinase to negatively regulate the type I interferon pathway by inhibiting the ubiquitination of retinoic acid-inducible gene I (RIG-I), TANK-binding kinase 1 (TBK1), and TNF receptor-associated factor 3 (TRAF3) [16]. In addition, SVA 3C^pro^ prevents the formation of stress granules (SGs) in infected cells, which are the sites of mRNA storage and involved in the regulation of mRNA translation by disrupting the interaction between eIF4GI and G3BP1 [17]. More recently, SVA 3C^pro^ was found to target nucleolin (NCL) and heterogeneous nuclear ribonucleoprotein A1 (hnRNP A1) to facilitate viral replication [18,19].

Various picornaviral 3C^pro^ structure-function studies showed that this family of cysteine protease shares a similar overall fold with many serine proteases, such as chymotrypsin and α-lytic protease [20–24]. These enzymes possess a conserved Cys-His-Asp/Glu catalytic triad at the active site that is similar to the Ser-His-Asp triad found in the vast majority of serine proteases. A second distinct function of picornaviral 3C^pro^ is the direct binding to viral RNA, and studies have shown that viral 3C^pro^ or its precursors can specifically bind to replication-linked cloverleaf RNAs, such as *oriL* and *oriR* in 5’- and 3’-noncoding regions [25,26]. These interactions are important for regulating viral replication and translation [27–30]. Moreover, Poliovirus (PV) 3C^pro^ was recently found to bind to phosphatidylinositol phosphate (PIP) in a region overlapped with the known RNA-binding site [31].

The crystal structure of SVA 3C^pro^ was recently reported (PDB ID: 6L0T) and its substrate specificity was characterized [32]. Notably, judging from the electron densities of catalytic Cys160 and His48, both residues should be assigned as alanines (S1 Fig). Therefore, this structure represents a double mutant (C160A/H48A) in an inactivated form, although this discrepancy has not yet been noted in the PDB database. In this work, we first report the crystal structure of wild-type SVA 3C^pro^, which indicates that SVA 3C^pro^ adopts a chymotrypsin-like fold and possesses the Cys-His-Asp catalytic triad conserved in most serine proteases. Unexpectedly, we identify an endogenous phospholipid molecule binding to a unique region, and indirectly characterize its effect on the protease activity and viral replication by mutations of lipid-binding residues. Our findings suggest that this phospholipid regulates proteolytic activity by acting as an allosteric activator. Our structure-function study provides a framework for future structure-based inhibitor design against this unique region in SVA 3C^pro^.

## Results

### SVA 3C^pro^ has a conserved catalytic triad and a variable β-ribbon

**Fig 1:**
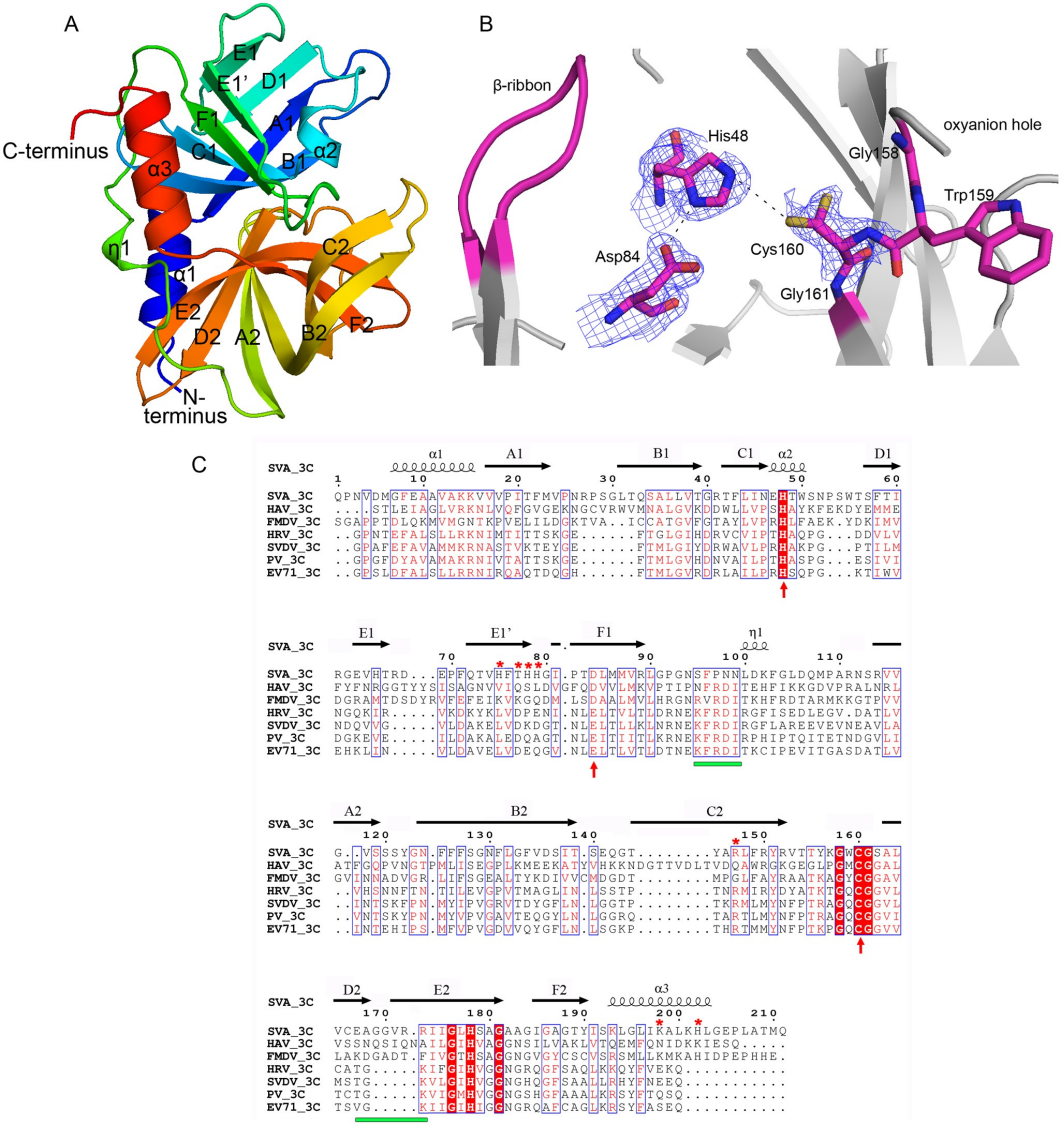
Overview of wild-type SVA 3C^pro^ structure. (A) Overall crystal structure of wild-type SVA 3C^pro^, colored from blue (N-terminus) to red (C-terminus). (B) The proteolytic site of SVA 3C^pro^. Unbiased omit electron density map (Fo-Fc) of the catalytic triad shown at a 1.5σ level. The key residues involved in catalysis and the β-ribbon involved in substrate specificity are highlighted in magenta. It is noted that Cys160 has an alternative conformation. (C) Structure-based sequence alignment of SVA 3C^pro^ with its representative homologs from various picornaviruses performed using clustal X (version 1.81) and ESPript 3. They include hepatitis A virus (HAV), foot-and-mouth disease virus (FMDV), human rhinovirus (HRV), swine vesicular disease virus (SVDV) and enterovirus 71 (EV71). The conserved residues are boxed in blue. Identical conserved and low conserved residues are highlighted in red background and red letters, respectively. The conserved catalytic triad (His48-Asp84-Cys160) and the residues involved in phospholipid-binding in SVA 3C^pro^ are highlighted using red arrows and red asterisks, respectively. The conserved phospholipid/RNA-binding motifs “K(R)F(V)RDI” and “V(T)GK” in most picornaviruses are labeled using green bars.

We first sought to solve the structure of wild-type SVA 3C^pro^ at 1.99 Å resolution by molecular replacement using the recently reported SVA 3C^pro^ mutant (C160A/H48A) structure (PDB ID: 6L0T) as the search model (Table 1). SVA 3C^pro^ is composed of two topologically equivalent antiparallel six-stranded β-barrel domains (A1-F1 and A2-F2, Fig 1A and 1C). The two domains are connected via a long loop (residues 90 to 111) mixed by the small helix η1. The two β-barrel domains pack together to form a shallow cleft for substrate binding (Fig 1A). SVA 3C^pro^ adopts a typical chymotrypsin-like fold that shows significant structural similarities with other picornaviral 3C^pro^ enzymes, such as those from foot-and-mouth disease virus (FMDV) (PDB ID: 2J92), Poliovirus (PV) (PDB ID: 2IJD) and hepatitis A virus (HAV) (PDB ID: 1QAV).

**Table 1:** X-ray Data collection and refinement statistics.

Data collection	SVA 3C^pro^ wild-type	SVA 3C^pro^ C160A
Beamline	SSRF 02U1	SSRF 02U1
Wavelength (Å)	0.9788	0.9788
Space group	*P*2_1_2_1_2_1_	*P*2_1_2_1_2_1_
Unit-cell parameters	a = 47.3 Å, b = 69.5 Å, c = 75.5 Å, α = β = γ = 90°	a = 47.3 Å, b = 69.5 Å, c = 75.5 Å, α = β = γ = 90°
Resolution (Å)	1.99 (2.11–1.99)^a^	1.61 (1.69–1.61)^a^
Number of unique reflections	25880 (4279)	32177 (4383)
Completeness (%)	100 (99.7)	97.0 (92.6)
Redundancy	35.9 (25.8)	12.0 (8.0)
Mean I/ ơ (I)	17.0 (1.8)	22.2 (2.7)
Molecules in asymmetric unit	1	1
R_merge_ (%)	13.1 (75.2)	5.5 (54.6)
R_meas_ (%)	13.3 (76.6)	5.9 (61.9)
CC_1/2_	100 (87.0)	99.9 (85.8)
**Structure refinement**		
Reflections used in refinement	17658	32104
Resolution range (Å)	40.10–1.99	27.92–1.61
R_work_/R_free_ (%)	21.3/24.9	17.5/19.3
Protein atoms	1599	1595
Protein residues	208	208
Waters	51	213
Average B factor (Å^2^)		
Protein	48.0	22.2
Ligand (CL)	51.2	
Ramachandran plot (%)		
Most favoured	96.1	96.6
Allowed	3.9	3.4
Disallowed	0	0
R.m.s. deviations		
Bond lengths (Å)	0.007	0.006
Bond angles (°)	0.932	0.875

^a^ the values in parenthesis means those for the highest resolution shell.

Three closely positioned residues (His48, Asp84 and Cys160) within the proteolytic active site of SVA 3C^pro^ comprise the canonical catalytic triad (Fig 1B). Cys160 and His48 may function as the nucleophile and the general acid ± base catalyst, respectively, while Asp84 may stabilize the resulting positive charge on His48. The catalytic important motif 158-GWCG-161, serves to position Cys160 for nucleophilic attack and to form the oxyanion hole with an electrophilic feature. Judging from the electron density of Cys160, the sulfhydryl group appears to have an alternate conformation (with an angle of ~120°orientation). The distance between the two sulfate atoms from two Cys160 possible conformations and His48 Nε2 is 3.7 Å and 4.7 Å in each conformation, respectively. Asp84 forms a hydrogen bond (H-bond, 3.2 Å) with His48 Nε2 for its stabilization. Structural superposition of different triads shows that SVA 3C^pro^ has a very similar configuration to those of other picornaviral 3C^pro^ enzymes (S2 Fig).

**Fig 2:**
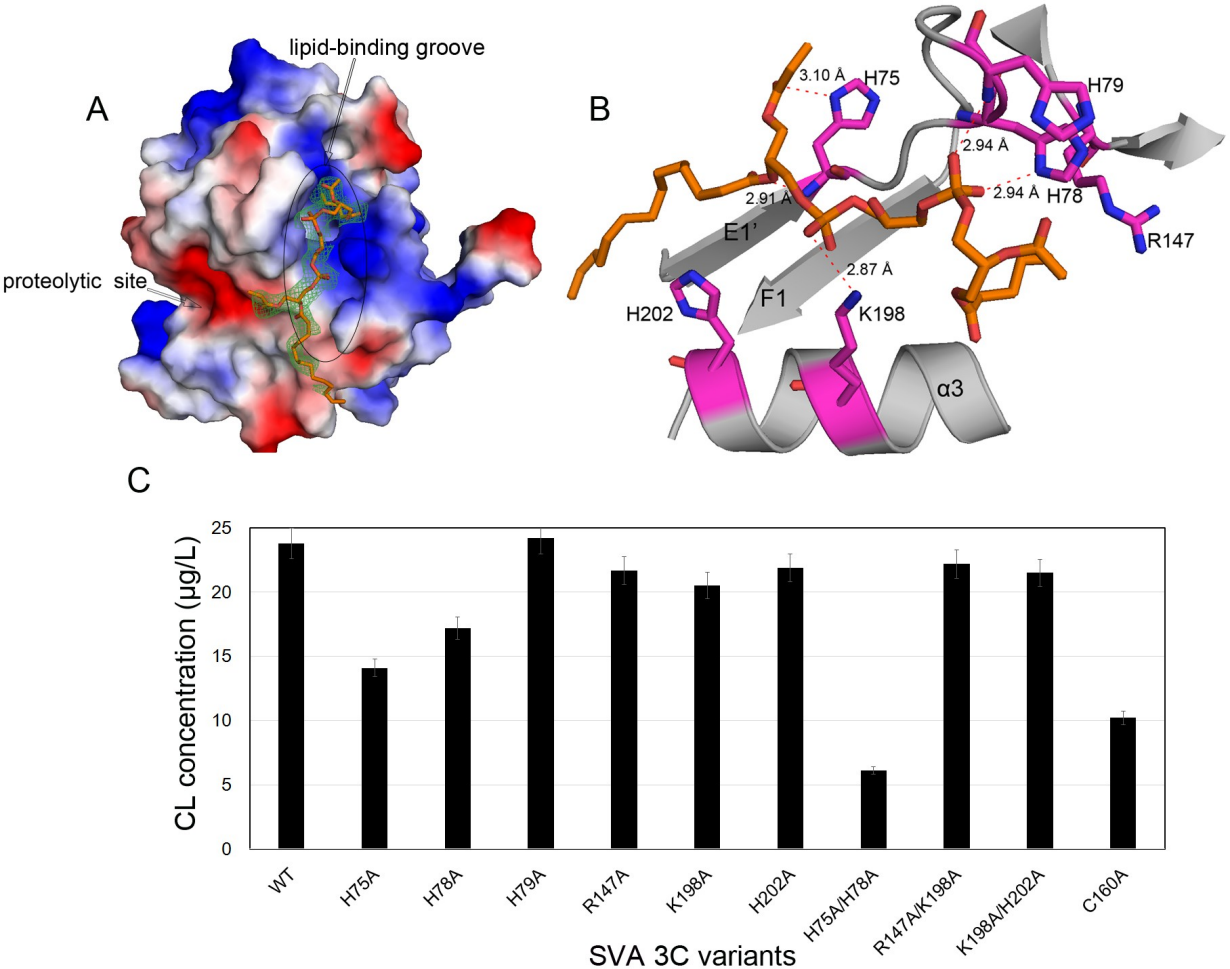
Identification of an endogenous phospholipid molecule in SVA 3C^pro^. (A) Close-up view of the surface charge surrounding the phospholipid-binding region of SVA 3C^pro^ (blue, +6.3KT; red, -6.3KT), colored by the local electrostatic potential. The region is predominantly electropositive. Electron density map (2Fo-Fc) of the phospholipid-like molecule (modeled as a CL molecule) is shown at a 1.5σ level. (B) Contacts analysis (H-bond and charge complementary) between the CL molecule (orange sticks) and the interacting residues (magenta sticks). (C) Mutagenesis studies on the residues involved in phospholipid-binding in SVA 3C^pro^. These residues were mutated to qualitatively determine the lipid content in these extracts using a CL-detection ELISA kit.

A β-ribbon is located between βB2 and βC2 in SVA 3C^pro^ (residues 136–147, Figs 1A and 2A). The β-ribbons in SVA and other picornaviruses have variable sequences and conformations (S2 Fig), which play an important role in recognizing different peptide substrates. Moreover, structural comparisons revealed a shift (~3.5 Å) of the apical tip of the β-ribbon pointing toward the protease active site in the wild-type compared to the C160A/H48A mutant (PDB ID: 6L0T) (S1B Fig). This shift brings the β-ribbon closer to the active site and is associated with the substrate-binding observed in the SVA 3C^pro^–substrate complex structure that we discuss below.

### Identification of a phospholipid molecule in SVA 3C^pro^

During the refinement of the SVA 3C^pro^ structure, we observed a portion of extra electron density located in the cleft mainly composed of E1’-F1 loop and α3 (Fig 2A and 2B). The region is composed of dominantly positive residues, including His75, His78, His79, Arg147, Lys198 and His202, which neighbor the proteolytic site. Considering the rough shape of this the non-protein electron density and the complementary charges, we considered this as a phospholipid-like ligand.

**Fig 3:**
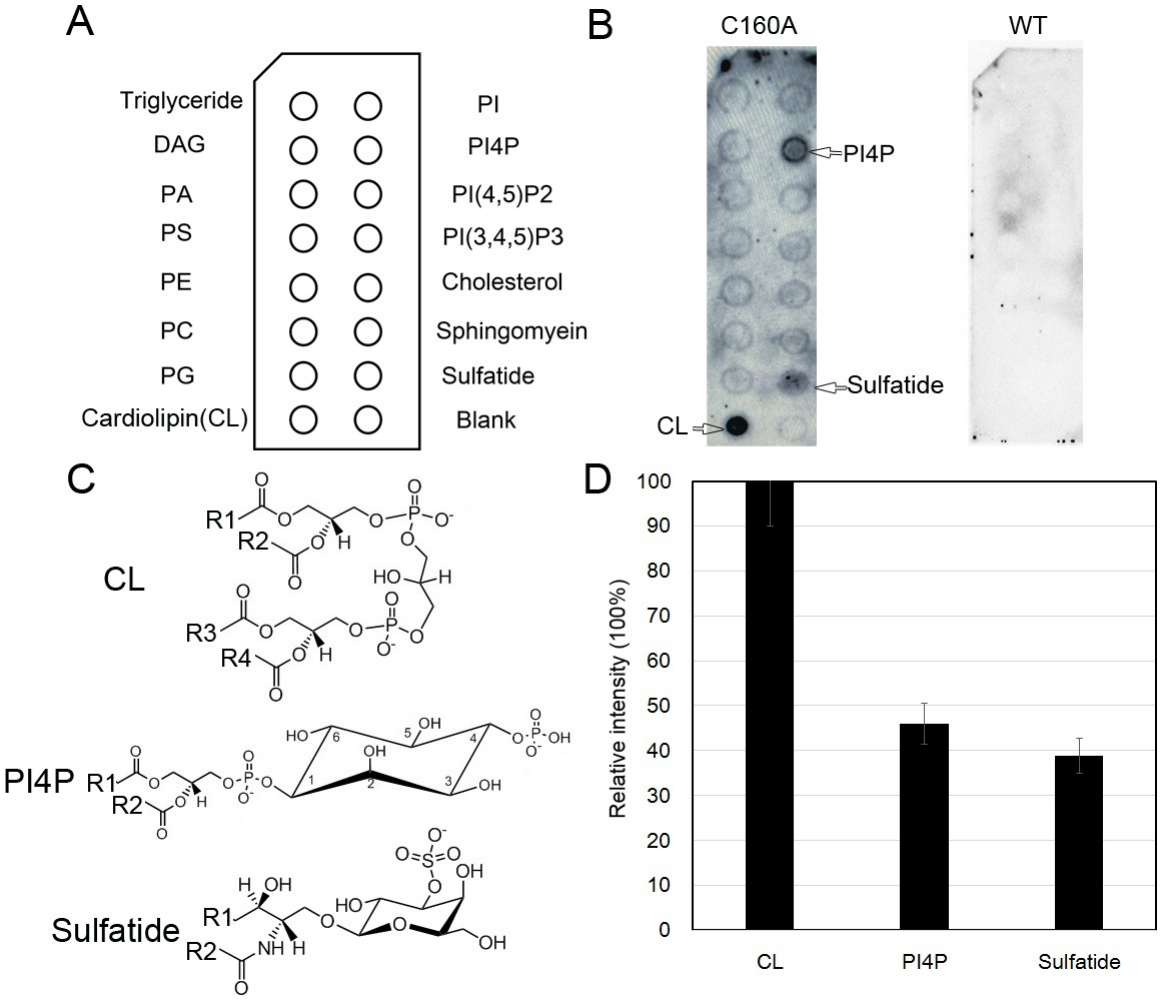
Identification of phospholipids categories in SVA 3C^pro^. (A) Membrane Lipid Strips (Echelon P-6002) contain the following lipids at 100 pmol per spot: Triglyceride, Diacylglycerol (DAG), Phosphatidic acid (PA), Phosphatidylserine(PS), Phosphatidylethanolamine (PE), Phosphatidylcholine (PC), Phosphatidylglycerol (PG), Cardiolipin (CL), Phosphatidylinositol (PI), Phosphatidylinositol 4-phosphate (PI4P), Phosphatidylinositol (4,5)-bisphosphate (PtdIns(4,5)P_2_), Phosphatidylinositol (3,4,5)-trisphosphate (PtdIns(3,4,5)P_3_), Cholesterol, Sphingomyelin and Sulfatide. (B) A lipid-binding assay of the extracts of SVA 3C^pro^ C160A and wild-type using the membrane lipid strip. Duplicate experiments were performed, and one representative blot was shown. The top three abundant lipids categories (CL, PI4P and sulfatide) are highlighted using arrows. Lipid strips were incubated with His-tagged SVA 3C^pro^ WT (10 μg/mL) or C160A (0.5 μg/mL). The strips were then washed and developed with a mouse anti-His antibody followed by anti-mouse IgG-HRP. (C) The chemical structures of CL, PI4P and sulfatide. R1/R2/R3/R4: fatty acid chain. (D) The relative intensities of CL, PI4P and sulfatide spots that represent their relative abundances in SVA 3C^pro^. The intensity of each spot was calculated using the software Image Lab. Data are presented as the average (±standard error of the mean) from duplicate experiments.

To verify the presence of the phospholipid-like molecule in SVA 3C^pro^, we investigated its lipid-binding capacity using a membrane lipid strip containing a variety of diverse phospholipids (Fig 3A). Unexpectedly, the wild-type protein showed no detectable binding to any of the tested categories of lipids, which could be explained by the lipid-binding region already being occupied by a phospholipid molecule. In the catalytically inactive C160A mutant structure, a phospholipid-like molecule could not be built due to lack of interpretable electron density data. (S3 Fig). In PV 3C^pro^, the protease activity and RNA/lipid-binding are mutually regulated and the two sites are dynamically coupled [33]. Similarly, we presumed that mutation of Cys160 may affect phospholipid-binding and partial phospholipids may be dynamically separated from the binding region; Therefore, the mutant could be used for phospholipid detection. As expected, the C160A mutant showed strong binding to cardiolipin (CL) as well as to phosphoinositol-4-phosphate (PI4P) and sulfatide (Fig 3B and 3C), but displaying weak bindings to other categories of phospholipids, such as phosphatidic acid (PA), phosphatidylserine (PS) and phosphatidylethanolamine (PE). By quantitatively analyzing the intensities of the three positive spots, we demonstrated that the binding intensity to CL is 2-3-fold that of PI4P and sulfatide (Fig 3D). Thus, CL is likely the dominant category of phospholipid that binds to SVA 3C^pro^.

**Fig 4:**
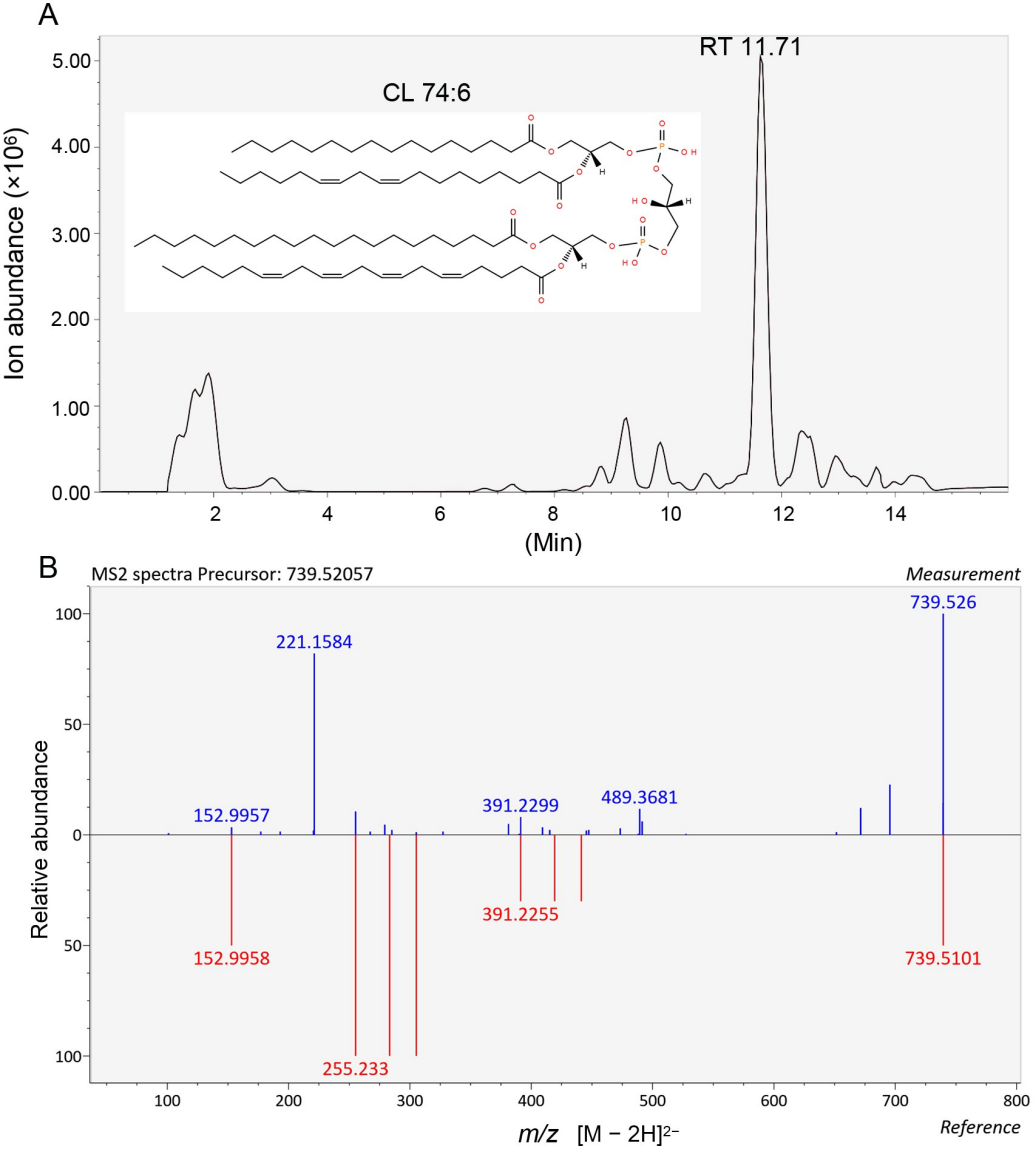
The untargeted lipidomics analysis of wild-type SVA 3C^pro^ extract by HPLC-MS. (A) The total ion chromatography (TIC) of lipid ions was recorded in the negative ion detection mode [M − 2H]^2−^. The chromatogram of phospholipid ions from accurate *m/z* values obtained from HPLC-MS spectra was used as input for a search in the lipid database LipidMaps. The search within CL category was accomplished by setting a mass tolerance of ± 0.01 *m/z* units. A CL molecule (CL 74:6) corresponds to a major peak in the TIC of lipid ions, with a retention time (RT) of 11.71 min. The chemical structure of CL 74:6 (CL 16:0_18:0_20:3_20:3) is derived from LipidMaps. (B) The MS^2^ spectra of the precursor *m/z* (upper panel) and the reference *m/z* (lower panel) for CL 74:6.

We further investigated the specific CL species in the wild-type SVA 3C^pro^ extract using HPLC-MS-based untargeted lipidomics. The total ion chromatography (TIC) of lipid ions was recorded in the negative ion detection mode (Fig 4A). For the untargeted lipidomics dataset, the precursor ion mass-to-charge ratios (*m/z* values) were matched to corresponding lipids using the LIPID MAPS structure database and LipidBlast at the sum composition. The detected CL type and composition recognition were performed using the relevant accurate *m/z* data (accuracy >5 ppm) as input for a search using the LIPID MAPS database. The MS^2^ spectra revealed two potential CL species (CL 74:6 and DLCL 21:1) in the SVA 3C^pro^ extract (S1 Data). The CL 74:6 species corresponds to a major peak in the TIC of lipid ions, with a retention time (RT) of 11.71 min, whereas the DLCL 21:1 species corresponds to a minor peak with the RT of 1.63 min (Fig 4A). The 74:6 species is thus probably the dominant CL with a precursor *m/z* of 739.52 that was well matched to that of a standard lipid (CL 74:6|CL 16:0_18:0_20:3_20:3) with a reference *m/z* of 739.51 [M − 2H]^2−^ (Fig 4B). It should be noted that PI4P was unable to be detected under the present HPLC-MS conditions, and we did not continue to pursue the potential PI4P species.

### Assignment of a CL molecule to a basic region of SVA 3C^pro^ structure

Based on the lipid-binding assay and lipidomics data, the CL 74:6 molecule (with a molecular composition of C_83_H_150_O_17_P_2_) was assigned to the identified electron density in SVA 3C^pro^. The CL molecule, especially its two phosphate groups, was well defined in the electron density map after refinement (Figs 2A and S3). The Ligplot analysis showed that the phosphate groups and the fatty acid chains of the CL molecule are mainly stabilized by several H-bonds and hydrophobic interactions, respectively (Figs 2B and S4). Moreover, the predominantly positive groove (mainly comprising His75/His78/Arg147/Lys198/His202), complements the two negatively charged phosphate groups for further stabilization (Fig 2A). Remarkably, a structure-based sequence alignment showed His75 and His78, which are closely related to the enzymatic activity of SVA 3C^pro^ and viral infectivity titers (data below), are distinct from the corresponding residues in other picornavirus 3C^pro^ (Fig 1C).

To identify the key residues involved in phospholipid-binding, the CL-interacting residues were single- or double-mutated and these mutants were purified. The lipids extracted from the same amount of mutant proteins were detected using a CL-specific enzyme-linked immunosorbent assay (ELISA) kit (Fig 2C). The lipid contents in H75A and H78A mutants were lower (~14.6 and ~18.1 μg/L, respectively) than that of the wild-type (~24.2 μg/L), whereas other single mutants (H79A, R147A, K198A and H202A) retained similar lipid-binding capacities relative to that of the wild-type. The H75A/H78A double mutant had the lowest lipid content (~5.8 μg/L), while two other double mutants (H79A/R147A and K198A/H202A) exhibited minimal changes in the lipid-binding capacities. In addition, the CL content in the C160A mutant was only ~40% of the wild-type, confirming that mutation of Cys160 affected lipid-binding and that the lipid could therefore not be modelled in C160A structure.

### SVA 3C^pro^ enzymatic activity may be positively regulated by the phospholipid

In order to investigate whether the phospholipid is associated with the protease activity, the enzymatic kinetics of purified SVA 3C^pro^ (wild-type, H75A and H78A) were studied using a fluorescence-labeled peptide from the SVA polyprotein 3C-3D junction as the substrate. The enzymatic kinetics data were well fitted to Hill equation that describes the cooperative binding of ligands, instead of Michaelis-Menten equation. Notably, the Hill coefficient value (*n*_H_) of the wild-type enzyme was 4.91, indicating a positive cooperativity (*n*_H_ > 1) of the phospholipid (Table 2 and S5A Fig). Furthermore, the H75A and H78A mutants, whose CL-binding capacities were notably decreased, had an *n*_H_ of 1.11 and 3.21, respectively. This indicates that mutation of the two residues (especially His75) may disrupt such cooperative binding (*n*_H_ = 1: no cooperativity). On the other hand, the catalytic efficiency (*k*_cat_/*K*_*half*,_ represents the intact protease activity in this study) of H75A and H78A were ~1.7- and ~1.4-fold of the wild-type, respectively. Thus, the proteolytic activity may be positively correlated with the lipid-binding capacity.

**Table 2:** Kinetic parameters of purified SVA 3C^pro^ wild-type and mutants calculated by Hill equation. Data are presented as the average (±standard error of the mean) from three independent experiments. A 95% confidence interval (CI) was provided for each parameter (*K*_*half*_, *V*_max_ and *n*_H_) in a bracket. The parameter *k*_cat_/*K*_*half*_ represents the protease activity of SVA 3C^pro^.

SVA 3C^pro^ variants	*K*_*half*_ (μM) (95% CI)	*V*_max_ (μM min^-1^) (95% CI)	*k*_cat_/*K*_*half*_ (μM^-1^min^-1^, 10^−3^) (95% CI)	*n*_H_ (95% CI)
**WT**	19.31 ± 2.88 (17.82–20.86)	0.20 ± 0.06 (0.19–0.21)	10.36 ± 2.35	4.91 ± 1.13 (3.82–6.17)
**H75A**	46.23 ± 4.56 (32.21–56.19)	0.29 ± 0.09 (0.22–0.51)	6.27 ± 2.44	1.11 ± 0.25 (0.67–1.86)
**H78A**	17.25 ± 3.96 (15.06–19.49)	0.12 ± 0.07 (0.11–0.13)	6.96 ± 1.29	3.21 ± 1.37 (2.03–4.87)
**H75A/H78A** ^a^	N.D.	N.D.	N.D.	N.D.

^a^ the mutant showed no detectable cleavage for the peptide substrate and its kinetic parameters were not calculated.

Unexpectedly, the H75A/H78A double mutant showed no detectable cleavage for the peptide substrate (Table 2 and S5B Fig), indicating the proteolytic activity is completely inhibited. As the lipid-binding capacity of the mutant is significantly decreased, the lipid-binding region may be necessary for the proteolytic activity. We were unable to completely remove the binding phospholipids in the purified wild-type SVA 3C^pro^ that was used for the kinetic studies despite various denaturation and renaturation attempts. Therefore, we cannot currently provide direct evidence to support such positive cooperativity between the phospholipid and the protease activity.

### Generation of an SVA 3C^pro^-substrate complex by crystal packing

**Fig 5:**
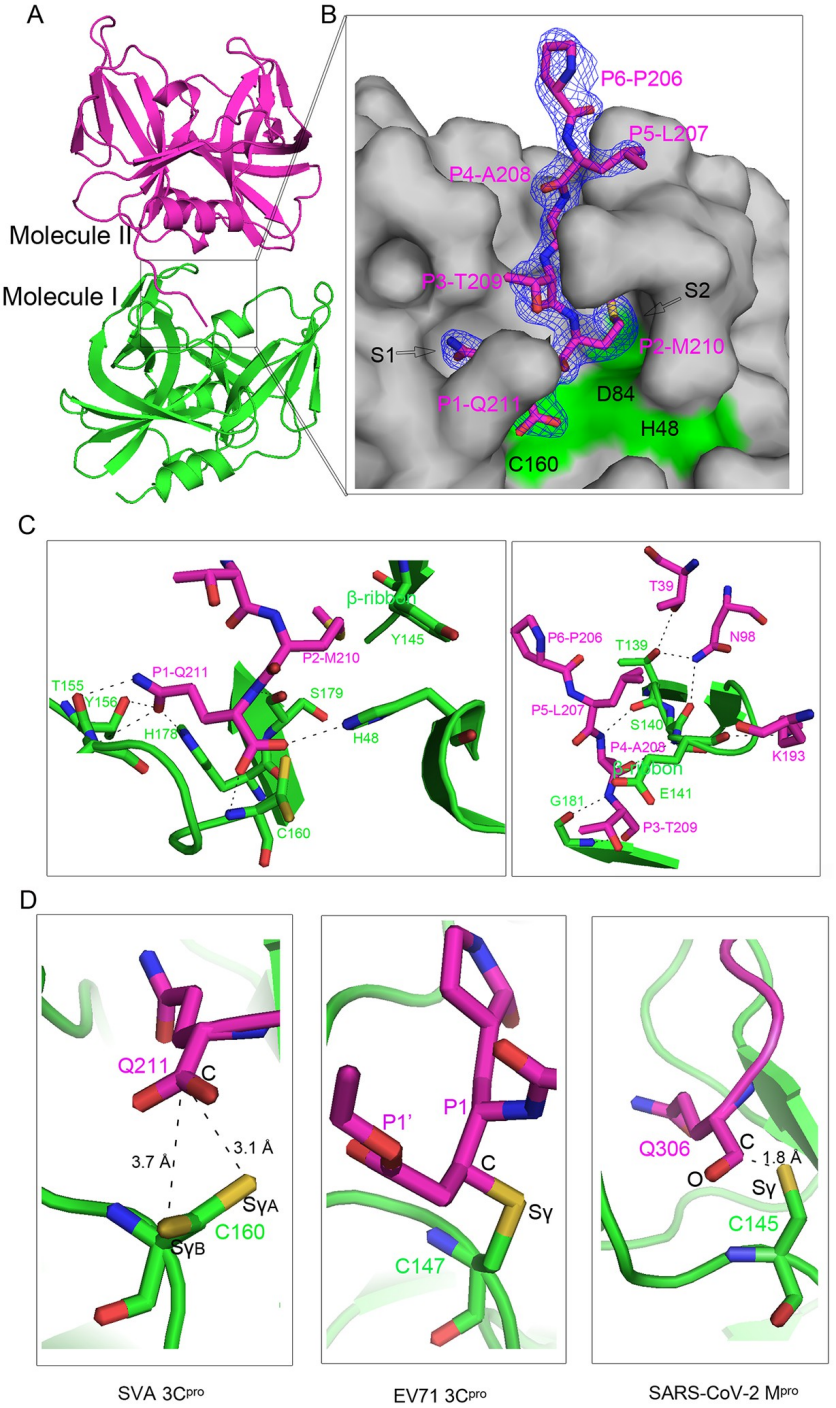
Crystallographic structure of SVA 3C^pro^ in complex with the physiological substrate. (A) Crystal packing of two protein molecules (Molecule I and II in green and magenta, respectively) that generate SVA 3C^pro^-substrate complex. (B) Close-up view of the C-terminus containing the autocleavage sequence (shown as magenta sticks) from Molecule II binding into the proteolytic active pocket from Molecule I (shown as surface). The catalytic triad is highlighted in green. Electron density map (2Fo-Fc) of the cleavage peptide is shown at a 1.5σ level. (C) Contacts analysis of the C-terminus of Molecule II with the active site of Molecule I. (D) Contacts analysis between substrate glutamate (or the inhibitor rupintrivir, magenta sticks) and catalytic cysteine (green sticks) in SVA 3C^pro^, EV71 3C^pro^ (PDB ID: 3SJO) and SARS-CoV-2 M^pro^ (PDB ID: 7KHP). The first two groups in rupintrivir binding by EV71 3C^pro^ are labelled using P1’ and P1, respectively.

Although there is a single molecule in the asymmetric unit, crystal packing analysis revealed the two neighboring protein molecules (named as Molecule I and II, respectively) have close contacts (Fig 5A). The oligomeric state study by analytical ultracentrifugation (AUC) showed that SVA 3C^pro^ has a sedimenting boundary at 23.5 kDa (very close to the theoretical molecular weight of 22.8 kDa) (S6 Fig), suggesting it is a monomer in solution as observed in other picornaviral 3C^pro^ enzymes [23]. Interestingly, the C-terminus containing the natural autocatalytic processing site (GEPLATMQG) from Molecule II, inserts into the proteolytic active site of Molecule I. Therefore, despite a crystallography artefact formed by the two contacting molecules, the SVA 3C^pro^-substrate complex likely captures the physiological peptide cleavage process of SVA 3C^pro^. According to the nomenclature of Berger and Schechter [34], the amino acids within each junction site are designated “P” or “P’” residues. The newly generated C terminus after the cleavage of the scissor bond is denoted P1, preceded by the P2, P3, etc., residues, and the N terminus yielded by cleavage is denoted P1’, followed by the P2’, P3’, etc., residues. Accordingly, those sites within the 3C protease that accommodate substrate “P” or “P’” residues are designated “S” or “S’” subsites.

The substrate peptide adopts an extended linear conformation with clear electron densities (Fig 5B). This peptide binds largely within the deep surface groove and contacts the protease via an extensive network of H-bonds and apolar interactions (Fig 5C). P1-Gln211 (Molecule II) is accommodated by the S1 specificity pocket, mainly composed of His178 and Thr155 (Molecule I). The oxygen atom in the side chain of Gln211 is stabilized by three H-bonds with Thr155, Tyr156 and His178 (2.6, 3.6 and 2.6 Å), whereas the nitrogen atom is stabilized by two H-bonds with Thr155 and Gly181 (3.0 and 3.3 Å). The nitrogen atom in the main chain of Gln211 is stabilized by a H-bonds with Ser179 (3.2 Å). In addition, one oxygen of the terminating carboxylate of P1-Gln211 forms a hydrogen bond (H-bond) with the main chain nitrogens of Cys160’ (3.3 Å). The second carboxylate oxygen (OXT) of P1-Gln211 is positioned to form a H-bond with His48 Nε2 (3.0 Å), which supports its role as a general acid ± base catalyst. P2-Met210 is located at the entrance of the S2 pocket and mainly makes hydrophobic interactions with Y145 from the β-ribbon (Fig 5B). Most residues at P2 position are hydrophobic (such as Met, Leu and Phe) in the cleavage sequences of SVA 3C^pro^ (S1 Table). The S2 pocket with enough size can accommodate the side chains of various hydrophobic residues for their stabilization via Y145. The direct contacts can also be observed between P3-Thr209 and Gly181 (3.1 Å), as well as P4-Ala208 and Thr139 and Glu141 from the β-ribbon (2.9 and 3.2 Å), which are likely associated with substrate specificity. Lastly, residues Thr39, Asn98, Gly181 and Lys193 also interact with the β-ribbon, which are also important for further stabilization of various substrates.

### An un-covalent binding between the substrate and catalytic residue in SVA 3C^pro^

In our SVA 3C^pro^–substrate complex structure, the main chain carbonyl carbon of the substrate Gln211 was 3.1 and 3.7 Å from each γ-sulfur atom (Sγ) in two alternative sulfhydryl groups of the Cys160 nucleophile (Fig 5D). These distances imply that it would be difficult for Cys160 to covalently bind to Gln211 through a thioester linkage. Until now, most reported picornavirus 3C^pro^ structures in complex with the substrate peptide have been obtained in settings where the nucleophile Cys was mutated to Ala to avoid the automatic cleavage [22,24]. Such structures fail to provide the details whereby the intact acyl-enzyme intermediate of 3C^pro^ is bound to the substrate. On the other hand, the catalytic Cys is usually covalently bound to the inhibitors in the structures of picornavirus 3C^pro^ with the peptide-mimicking inhibitors [24,35], such as rupintrivir-binding EV71 3C^pro^ (Fig 5D). A recent study on SARS-CoV-2 main protease (M^pro^) structure showed that the C-terminus of one molecule can insert into the catalytic site of another symmetry-related molecule to generate a M^pro^-substrate complex [36], in a similar manner to our SVA 3C^pro^. The catalytic Cys145 is covalently bound to substrate Gln306 in the C-terminal autocleavage sequence (Fig 5D), representing its acyl-enzyme intermediate state. Meanwhile, non-covalent binding was observed between Gln306 and C145A in the mutant structure, which may represent the cleavage product-enzyme complex.

### The lipid-binding residues in 3C^pro^ contribute to the marked decrease in SVA infectivity titers

**Fig 6:**
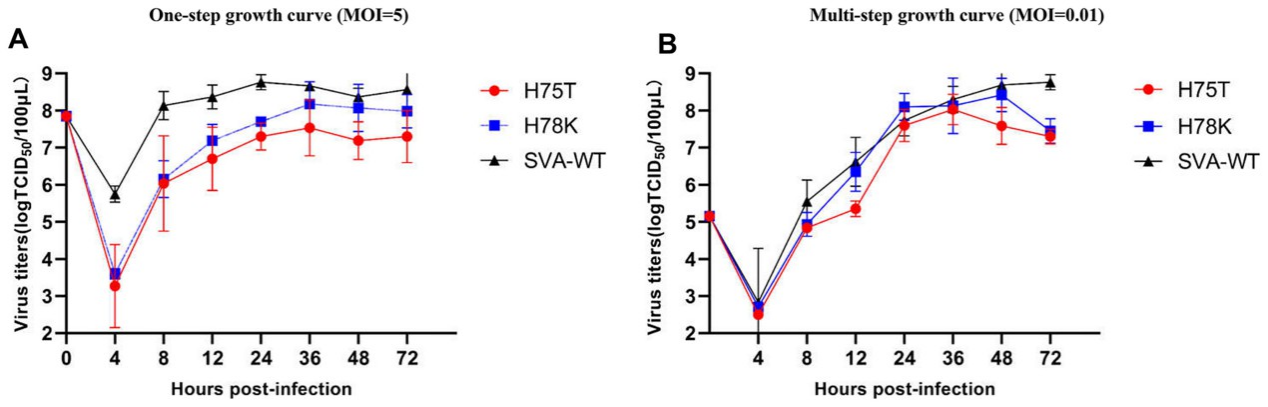
One-step and multi-step growth curves each mutant and SVA-WT. (A-B) BHK-21 cells were infected with mutant or WT virus at an MOI of 5 or 0.01, respectively. The resulting virus was harvested at different times, titered, and expressed as a TCID_50_ dose. The mean values ± SD (repeated measures ANOVA, n = 3, no significant differences identified) are shown.

The viral titers of SVA harboring the lipid-binding mutants (H75T/H78K) in 3C^pro^ were ~10-fold lower than that of the wild type, indicating a positive regulation of the lipid on SVA infection capacity. The His-to-Ala mutation at position 75 or 78 of 3C^pro^ reduced the CL-binding capacity of H75A or H78A relative to wild-type 3C^pro^. Thus, we hypothesized that the phospholipid-binding capacity to the 3C^pro^ affects SVA replication. However, neither of the SVA mutants (rH75A or rH78A) failed to be rescued, whereas viable SVA mutants rH75T or rH78K were rescued. One-step growth curves showed that the replication of the mutants were ~10-fold lower than that of the SVA-WT (Fig 6A), indicating that the single amino acid mutant (H75T) affects viral replication. However, multi-step growth curves showed that the mutants exhibited replication kinetics similar to that of the SVA-WT after infection of BHK-21 cells at a low multiplicity of infection (Fig 6B). Our results suggest that the phospholipid-binding region in 3C^pro^ is not only related to protease activity but also closely related to virus replication.

## Discussion

In this study, by the combination of crystallographic structure, untargeted lipidomics and immune blot, we identified an endogenous phospholipid molecule that binds to SVA 3C^pro^. Importantly, analysis of the enzymatic kinetics indicated a positive cooperativity between the phospholipid-binding and proteolytic activity, which is similar to the cooperative binding of the ligands observed in hemoglobin and other allosteric proteins [37]. The proteolytic activity of wild-type SVA 3C^pro^ was activated in the presence of the lipid, whereas the activity is inhibited when the lipid-binding capacity was decreased. The endogenous phospholipid molecule may therefore function as an allosteric activator to regulate SVA 3C^pro^ proteolytic activity. Moreover, mutations in the lipid-binding residues (H75T/H78K) could significantly reduce the infectivity titer of SVA, suggesting that phospholipid-binding plays an important role in the regulation of virus replication. On the other hand, 3C^pro^ plays multiple roles in viral replication and infection, such as cleavage of viral polyprotein/host proteins and viral genomic RNA-binding besides lipid-binding. Therefore, it is complicated to show whether these factors work together with the lipid-binding to affect SVA replication, and we cannot currently provide direct evidence to prove it.

The two phospholipid/RNA-binding motifs K(R)FRDI and VGK are highly conserved in most picornaviral 3C^pro^ enzymes and play an important role in picornaviral RNA replication [38,39]. The conserved K(R)FRDI motif forms a basic patch that sits on the face opposite the protease active site (S7 Fig). This spatial relationship means that the RNA-binding site and the protease active sites may be allosterically affected by each other [30]. Unexpectedly, the two RNA/lipid-binding motifs correspond to 95-SFPNN-99 and 167-EAG-169 in SVA 3C^pro^ (Fig 1C), respectively, which are likely not involved in RNA-binding. Instead, the phospholipid-binding region is mainly composed of the motifs 75-HFTHH-79 and 198-KALKH-202 in SVA 3C^pro^ (S7 Fig). Unlike the phospholipid/RNA binding regions that are located on the face opposite from the active site in most picornaviral 3C^pro^ enzymes, this basic region is located adjacent to the protease active site for potential RNA-binding. Therefore, the phospholipid binds to a unique region of SVA 3C^pro^ distinct from those of most picornaviruses.

Several functional and structural studies have suggested the interrelation of the two functions of picornaviral 3C^pro^ [39]. A previous study showed that mutation of the RNA-binding site was found to affect both RNA-binding and proteolytic activities in EV71 3C^pro^ [30]. The study using nuclear magnetic resonance spectroscopy (NMR) and small-angle X-ray scattering (SAXS) studies of rhinovirus 3C^pro^ suggested that RNA-binding may induce subtle conformational changes in the proteolytic active site [40]. A further study on PV 3C^pro^ using NMR and molecular dynamics simulations (MD) showed that binding of peptide and RNA induced structural dynamics changes at both the protease active site and the RNA-binding site [33]. The following functional study revealed that protease activity and RNA-binding are mutually affected, suggesting an allosteric control of the two functions with potential cooperative effects. In this study, the proteolytic activity of wild-type SVA 3C^pro^ was shown to be activated in the presence of the lipid, and the activity was decreased (H75A and H78A) or completely inhibited (the double mutant) when lipid-binding capacity was disrupted. The enzymatic kinetic suggested a cooperative binding model that describes the correlation between the proteolytic activity and the lipid-binding capacity. The phospholipid may thus act as an allosteric activator to regulate SVA 3C^pro^ proteolytic activity and virus replication. The corresponding phospholipid-binding region may thus function as an allosteric site, which is located between the two topologically equivalent domains of SVA 3C^pro^ (S7 Fig). We suppose that the lipid-binding may stimulate the proteolytic activity by inducing the conformation change of the active site, although we were unable to obtain lipid-free SVA 3C^pro^ structure. On the other hand, the conformation change of the active site may also affect the lipid/RNA-binding capacity to achieve the cooperativity.

**Fig 7:**
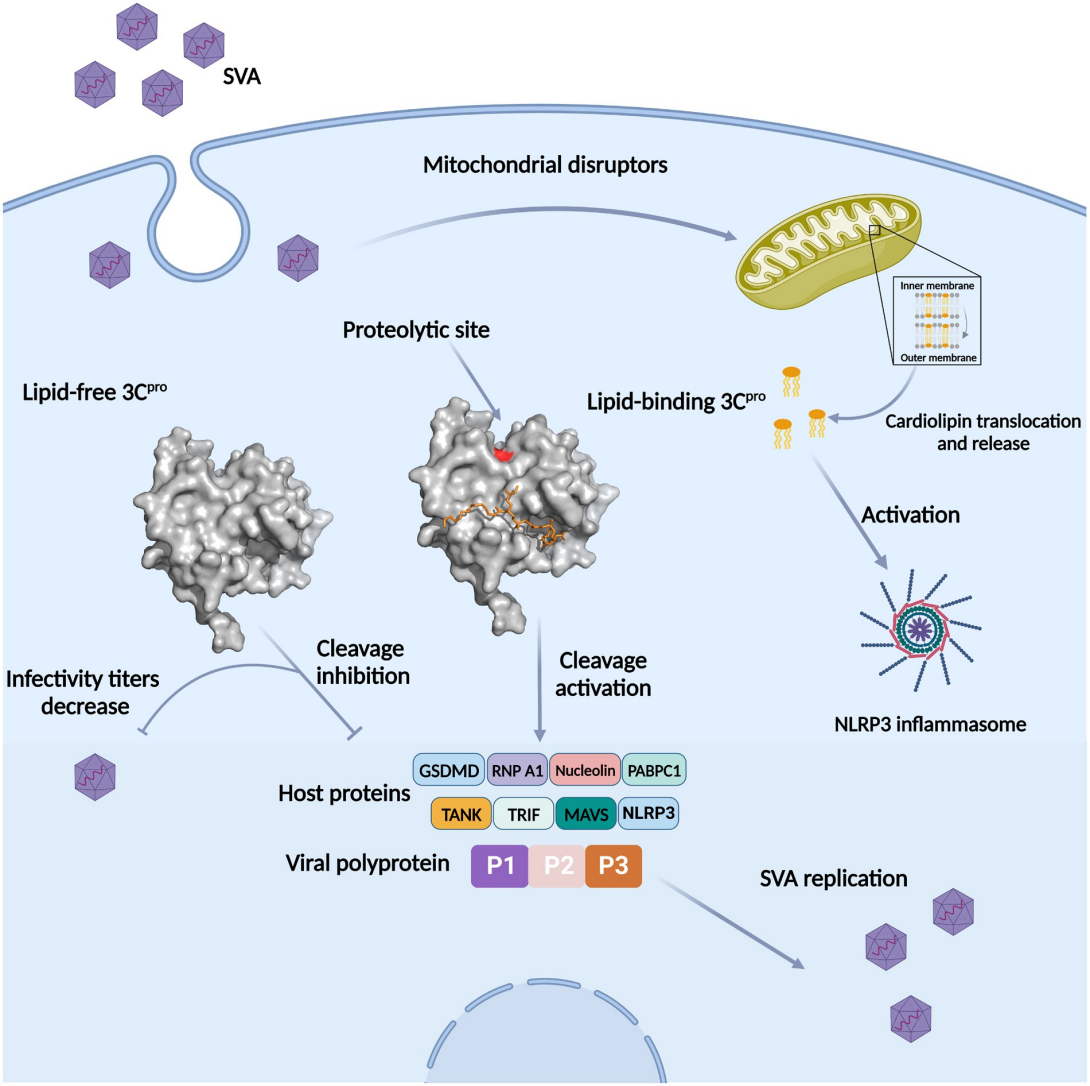
A supposed mechanism on the regulation of SVA 3C^pro^ proteolytic activity and viral infection capacity by an endogenous Cardiolipin (CL). SVA infection may cause inflammatory insults and subsequent partial CL redistribution from the inner membrane to the cytoplasm-facing outer membrane of mitochondria. In the cytoplasm, SVA 3C^pro^ binds to the CL in a unique region neighboring the proteolytic site. The lipid can activate its cleavage activity to ensure its efficient replication. Meanwhile, the infectivity titer of SVA harboring the lipid-binding mutants in 3C^pro^ are significantly lower than the wild-type. In the lipid-free status of SVA 3C^pro^, its enzymatic activity is inhibited and the cleavage capacity for the viral polyprotein and several host proteins is decreased. The phospholipid may function as an allosteric activator to regulate SVA 3C^pro^ proteolytic activity, and as a switch to keep a balance between replication and infection.

The lipid-binding assay showed that SVA 3C^pro^ C160A has a strong preference for CL, followed by PI4P and sulfatide, suggesting SVA 3C^pro^ possesses a broad lipid-binding activity. The untargeted lipidomics analysis further revealed a dominant CL molecule (CL 74:6) in SVA 3C^pro^. As a signature phospholipid in the inner mitochondrial membranes of eukaryotic cells, CL is required and sufficient for the activation of NLRP3 inflammasome by its direct binding to NLRP3 [41]. Inflammatory insults resulting from viral infection (or other mitochondrial disruptors) cause CL translocation from the inner to the outer membrane of mitochondria by membrane permeability transition [42]. Based on the literature and our data, we hypothesize a molecular mechanism on the regulation of SVA 3C^pro^ proteolytic activity and viral infection capacity by the CL (Fig 7). In our supposed model, SVA infection may cause partial CL redistribution from the inner to the outer membrane of mitochondria and subsequent release into the cytoplasm. The CL may allosterically activate SVA 3C protease activity for the cleavage of viral polyprotein as well as host proteins (such as NLRP3 and MAVS) to subvert host responses and ensure viral replication (Fig 7). A recent study on the subcellular distribution study in 293T cells showed that, whereas the wild-type 3C^pro^ was solely localized in the cytoplasm, the C160A/H48A mutant was partly distributed in the outer membrane of the mitochondria, and this may be related to the proteolytic activity that induces apoptosis [43]. The preferred binding of SVA 3C^pro^ to the CL may therefore explain the observation that a portion of C160A mutant can traffic to the mitochondria. The cytoplasmic CL could therefore bind and activate the NLRP3 inflammasome and the subsequent interleukin-1β (IL-1β) secretion.

In this study, we also found that SVA 3C^pro^ has a preferred binding to PI4P, a lipid that is produced by phosphatidylinositol-4-kinase IIIβ (PI4KIIIβ) and enriched in cellular membranes. The replication of most positive-strand RNA viruses requires remodeling of the cell membrane to convert this into viral replication organelles (ROs) [44–47]. The PI4P-rich lipid microenvironment within the ROs is essential for enteroviral RNA replication, whereby PI4P can bind to RNA polymerase (3D^pol^) to regulate its activity [48]. A recent study showed that ectopic expression of PV 3CD is sufficient to induce synthesis of multiple phospholipids, including PI4P, PIP2 and PC [49]. The 3CD can further hijack their biosynthetic pathways to serve as a master regulator of cellular phospholipid and membrane biogenesis. Another study also showed PV 3C^pro^ has a broad and specific PIP-binding activity to phospholipids, such as PI4P and PI(4,5)P_2_, and its binding to membranes is determined by PIP abundance [31]. Here, the preferred binding of SVA 3C^pro^ to PI4P suggests that it may help to recruit SVA 3C^pro^ (or 3CD) to the ROs and induce membrane biogenesis. We hypothesize that after SVA infection, PI4P-rich ROs are produced and that the PI4P-rich lipid microenvironment facilitates replication with the viral genome. On reaching a specific concentration, PI4P could then bind to the phospholipid-binding region of 3C^pro^ to regulate its activity.

Herein, we provide the first structural evidence supporting the regulation of picornaviral 3C^pro^ protease activity by endogenous phospholipids. Our results show that a unique phospholipid-binding region in SVA 3C^pro^ is fundamental for its proteolytic activity and virus replication. The phospholipid may function as a switch between replication and infection to maintain the balance between the status of proteolytic activity and inactive mutants. Our findings suggest that, in addition to the proteolytic active site, the phospholipid-binding region of 3C^pro^ in picornaviruses represents a novel key region that determines replication efficiency, which can be used for development of inhibitors to inhibit virus replication.

## Materials and methods

### Cloning

The optimized full-length DNA sequence encoding wild-type SVA 3C^pro^ was synthesized by Genscript Corp. (Nanjing, China) and cloned into the modified pET28a-SUMO (Novagen, USA) in which the cleavable ubiquitin-like-specific protease 1 (ULP1) fused with N-terminal His_6_ tag was introduced. The recombinant plasmid was transformed into *E*. *coli* DH5α cloning strain and plated onto LB-kanamycin plates. The plasmid was isolated and transformed into an *E*. *coli* BL21 (DE3) star expression strain (Invitrogen). Site-directed mutagenesis of SVA 3C^pro^ was performed by a PCR-based technique according to the QuikChange site-directed mutagenesis strategy (Stratagene) following the manufacturer’s instructions. The mutant genes were sequenced and found to contain only the desired mutations. The primers used in this study are shown in S2 Table.

### Protein expression and purification

Bacterial cells expressing wild-type SVA 3C^pro^ were grown to mid-log phase in LB media at 37°C in the presence of 50 μg/mL kanamycin. Induction of protein expression was initiated by adding isopropyl-1-thio-β-D-galactopyranoside (IPTG) to the culture to a final concentration of 0.4 mM, and the cells were grown at 16°C. Cells were pelleted after 20 h by centrifugation at 6000 *g* for 10 min at 4°C. The cell pellet was resuspended in a buffer containing 20 mM Tris (pH 8.0), 100 mM NaCl, 2 mM β-mercaptoethanol and 1 mM PMSF, and lysed by ultrasonication on ice. The cell debris and membranes were pelleted by centrifugation at 20 000 *g* for 60 min at 4°C. The soluble N-terminally His-SUMO-tagged SVA 3C^pro^ was purified by affinity chromatography with nickel-nitrilotriacetic acid resin (Bio-Rad, USA) and the tag was removed by protease ULPI overnight hydrolysis and reloaded with 20 mM imidazole. SVA 3C^pro^ was further purified by gel filtration (Superdex 75, GE Healthcare, USA) equilibrated in a buffer containing 20 mM Tris (pH 8.0), 300 mM NaCl and 2 mM DTT using an ÄKTA Purifier System (GE Healthcare, USA). Highly purified protein fractions were pooled and concentrated. Protein concentrations were determined using the Bio-Rad protein assay kit and crystallization trials were performed by ultrafiltration in an Amicon cell (Millipore, USA). All the SVA 3C^pro^ variants were expressed and purified following the same procedures (including the same buffers) to the wild-type protein.

### Protein crystallization

The initial crystallization conditions for SVA 3C^pro^ wild-type and C160A mutant (both are ~10 mg/mL) were obtained through the utilization of several sparse matrix screens (Emerald BioSystems, USA) with the sitting drop vapor diffusion method at room temperature after 14 days. The crystal quality was optimized by adjusting the concentration of the precipitant and pH value. The best crystal for SVA 3C^pro^ wild-type was obtained in solution containing 20% (w/v) polyethylene glycol (PEG) 3,350, 1% (w/v) tryptone and 0.05% (w/v) sodium azide. The best crystal for SVA 3C^pro^ C160A mutant was obtained in a solution containing 4% (w/v) PEG 8,000 and 0.1 M HEPES (pH7.5).

### Data collection, crystal structure determination and refinement

The diffraction data from a single crystal were collected on the beamline station BL02U1 of SSRF (Shanghai Synchrotron Radiation Facility) using an EIGER pixel detector at a wavelength of 0.9788Å. The total oscillation was 360° with 1° per image and the exposure time was 0.3 s per image. Before data collection, the crystals were soaked in the reservoir solution supplemented with 20% (v/v) ethylene glycol for a few seconds and then flash-frozen in liquid nitrogen. All the data were processed by the program HKL2000 [50]. The initial phases were calculated using the program PHASER [51] with the previously reported SVA 3C^pro^ structure (PDB ID: 6L0T) as the searching model. The structure was refined with the program Phenix.refine [52] and manually corrected in Coot [53]. The qualities of the final models were validated with the program MolProbity [54]. Refinement statistics and model parameters are given in Table 1. The program PyMOL (http://www.pymol.sourceforge.net/) was used to prepare structural figures. The program LigPlot^+^ [55] was used to check the hydrogen bond (within 3.8 Å) and hydrophobic interactions (within 5.0 Å) between the protein and lipid molecule.

### Lipid-binding assay

To assess the direct binding of SVA 3C^pro^ to lipids, a lipid-binding assay was performed using Membrane Lipid Strips (P-6002, Echelon Biosciences) according to the manufacturer’s instructions. In brief, the strip was blocked in TBST (50 mM Tris-HCl, 150 mM NaCl and 0.1% Tween 20, pH7.0) containing 5% fatty acid-free BSA (Sigma-Aldrich) for 1 h at room temperature in the dark followed by overnight incubation with recombinant SVA 3C^pro^ wild-type (10 μg/mL) or C160A (0.5 μg/mL) in blocking buffer at 4°C with gentle agitation. After washing membranes 3 times for 30 min using TBST, the strip was incubated for 1 h with anti-His tag monoclonal antibody (Cat No. HT501, TransGen Biotech, China). The strip was then incubated with HRP-conjugated secondary antibody (Cat No. HS201, TransGen Biotech, China). Finally, the spots were detected using an enhanced chemiluminescence (ECL) system (Cat No. 32016, Thermo Fisher Scientific) in Tanon 4200 Multi (Biotanon, Shanghai, China) after washing three times again with TBST.

### LC-MS based untargeted lipidomics for phospholipid identification in SVA 3C^pro^

The purified SVA 3C^pro^ (~ 0.1 mg/mL) from *E*. *coli*-expression was heated at 50°C for 30 min and the precipitation was centrifuged at 12000 *g* for 15 min at 4°C. The supernatant (100 μL) was transferred to a 1.5-mL Eppendorf safe-lock tube and mixed with 1 mL methyl tert-butyl ether. The tube was vortexed for 1 h, and the solution was transferred to a 1.5-mL Eppendorf safe-lock tube and mixed with 300 μL H_2_O. The sample was centrifuged at 12000 rpm for 10 min. The lipid extract (0.8 mL) in the methyl tert-butyl ether phase was dried in a vacuum concentrator and re-dissolved in isopropanol: acetonitrile solution (1:1). The sample was centrifuged at 12000 rpm for 10 min, and the supernatant (80 μL) was taken out for LC-MS detection.

A Nexera UHPLC LC-30A (Shimadzu Company, Japan) equipped with a nanoACQUITY system (Waters) coupled to TripleTOF5600+ (AB SCIEX) mass spectrometer equipped with an electrospray ionization (ESI) source was used. A volume of 10 μL of lipid extract was injected onto an ACQUITY XBridge BEH C18 Column (2.5 μm, 4.6 mm×150 mm) for chromatographic separation. Mobile phases consisted of (A) acetonitrile:H_2_O (6:4), 0.1% (v/v) formic acid in acetonitrile and 5 mM ammonium acetate, and (B) isopropanol: acetonitrile (9:1), 0.1% (v/v) formic acid in acetonitrile and 5 mM ammonium acetate. The initial condition was 20% B, maintained for 1 min. The mobile phase was ramped to 100% B from 1 min to 13 min, maintained at 0% A from 13 min to 16 min, ramped to 20% B from 3.5 min to 3.6 min, and maintained at 90% B from 3.6 min to 5 min. The column flow was 300 μL/min, and the column temperature was maintained at 40°C.

The raw MS data were imported to be processed by MS-DIAL software (version 4.70) for lipid identification [56]. The retention time (RT), precursor mass/charge ratio (*m/z*), isotopic ratios, collision cross-section (CCS) and MS/MS spectrum of the peaks were calculated. Lipid annotations were performed manually using the LIPIDMAPS structural database (LMSD) bulk structures and LipidBlast software [57] for positive mode [(M+H)+ ions] and for negative mode [(M-H)- ions]. A *m/z* error of 5ppm (Lipidmaps) or 0.008 Da (Lipidblast) was used.

### ELISA for quantitative determination of CL in in SVA 3C^pro^

The purified SVA 3C^pro^ wild-type and variants were heated and the precipitation was centrifuged as above. The lipid content in each sample was determined using a CL- or PI4P-specific enzyme-linked immunosorbent assay (ELISA) kit (Shuhua Bio, Shanghai, China), following the manufacturers’ instructions. The ELISA 48-well plates were pre-coated using anti-CL or anti-PI4P primary antibodies and were washed five times with TBS-T buffer. The samples or the standards were added into the plates and incubated for 30 min at 37°C. The plates were washed five times with TBST and HRP-labelled secondary antibodies were added and incubated at room temperature for 30 min at 37°C.

Following incubation, plates were washed four times in TBST and developed with Sure Blue Reserve TMB Microwell Peroxidase Substrate (KPL) for 10 min at 37°C. The reaction was stopped by the addition of TMB Stop Solution and the absorbance at 450 nm was measured using an ELISA plate reader.

### Protease activity assay

The synthetic fluorogenic peptide substrate Dabcyl-EPLATMQGLMTELE-Edans was attached with a fluorescence quenching pair Dabcyl and Edans, corresponding to SVA 3C–3D junction. Fluorescence experiments were performed with Tecan Spark 10M Multimode Plate Reader (Switzerland) at 30°C. The reaction mixture (100 μL) contains 50 mM Tris–HCl (pH 8.0), 200 mM NaCl, 2 mM DTT, 1 μM SVA 3C^pro^ or its mutants and fluorogenic peptides of different concentrations (15–120 μM) in a 96-well plate (Greiner). The instrument was pre-calibrated by Edans standard to calculate the relationship between relative fluorescence unit and concentration. The relative fluorescence unit (RFU) was collected using an excitation wavelength of 360 nm and by monitoring the emission 490 nm and was then converted to substrate concentration. The initial velocities of the reactions are plotted versus substrate concentrations. Values are averaged from three independent experiments. The data were fitted to Hill equation (below) to calculate *K*_*half*_, *V*_*max*_, *k*_cat_/*K*_*half*_ and Hill coefficient (*n*_H_) (including a 95% confidence interval for each parameter) using GraphPad Prism version 9.0 (San Diego, California USA).

$$V=\frac{V_{max}{\left[S\right]}^{n_{H}}}{{\left[K_{half}\right]}^{n_{H}}+{\left[S\right]}^{n_{H}}}$$

Where *V* is the reaction velocity; *V*_*max*_ is the maximum velocity of the reaction; *S* is the substrate concentration; *K*_*half*_ is the half-maximal concentration constant; *n* is the Hill coefficient that describes the cooperativity (*n*_H_ > 1: positive cooperativity; *n*_H_ < 1: negative cooperativity; *n*_H_ = 1: no cooperativity).

### TCID_50_ assay and virus growth curves

Ten-fold serial dilutions of viruses were prepared in 96-well round-bottom plates in Dulbecco’s Modified Eagle’s medium (DMEM), and 50μL of the dilution was transferred to 10^4^ BHK-21 cells plated in 100 μL of DMEM with 2% FBS. TCID_50_ (50% Tissue Culture Infectious Dose) values were determined by the Reed-Muench formula. Viral replication kinetics was performed as follows. BHK-21 cells in 6-well tissue culture plates were washed with phosphate-buffered saline (PBS) and inoculated with SVA-WT or SVA mutant viruses at the indicated multiplicity of infection (MOI). The plates were incubated for 1h at 37°C. The infected cells were washed three times with PBS to remove unbound virus particles and covered with DMEM containing 2% FBS with or without IMP-1088. The infected cells were incubated at 37°C and harvested at indicated times. After three consecutive freeze-thaw cycles, the viral titers were determined by TCID_50_ assay.

### Analytical ultracentrifugation (AUC)

The sedimentation velocity measurements were carried out using a Beckman Optima XL-I analytical ultracentrifugation (Beckman-Coulter Instruments) with a Ti rotor at 20°C. The purified SVA 3C^pro^ concentration was adjusted to absorption of ~0.8 at 280 nm. The SEDFIT program was used to analyze the sedimentation coefficient [58].

## Supporting information

S1 Fig Structural comparison of SVA 3C^pro^ in this study with the recently reported one (PDB ID: 6L0T). (A) Electron density map (2Fo-Fc) of the catalytic triad in PDB 6L0T, shown at a 1.5σlevel. It should be noted that Cys160 and His48 should be assigned as alanines judging from the electron densities. (B) Structural superposition of the active site between our SVV 3C^pro^ (light gray) and PDB 6L0T (dark gray). The catalytic triad and β-ribbon are highlighted in magenta and green, respectively.
(TIF)

S2 Fig A conserved proteolytic active site in SVA 3C^pro^. Structural superposition of the active site of SVV 3C^pro^ with those of representative homologs FMDV (PDB ID: 2J92) and HAV (PDB ID: 1QA7).
(TIF)

S3 Fig Comparison of the electron density in the lipid-binding region of wild-type structure with that in C160A mutant structure. Electron density maps (2Fo-Fc) in both structures are shown at a 1.5σ level in Coot.
(TIF)

S4 Fig The contacts analysis between the CL molecule and SVA 3C^pro^ by LigPlot^+^. The H-bonds hydrogen-bonds (H-bonds) and hydrophobic contacts are calculated within 3.8 Å and 5.0 Å, respectively.
(TIF)

S5 Fig Determination of SVA 3C^pro^ enzyme activities. (A) The plots of initial rate (v) *versus* the substrate peptide concentration for wild-type (WT) and variants. See Materials and Methods for experimental details and Table 2 for the *k*_cat_/*K*_*half*_ values determined for each variant. (B) Time course analysis of relative fluorescence unit (RFU) of the fluorogenic peptide cleaved by WT and H75A/H78A mutant.
(TIF)

S6 Fig Oligomeric state of recombinant SVA 3C^pro^ in solution by AUC. (TIF)

S7 Fig Structural comparison of the phospholipid-binding region in SVA 3C^pro^ with that in EV71 3C^pro^ (PDB ID: 3OSY). The phospholipid-binding motifs 75-HFTHH-79 and 198-KALKH-202 are located neighboring the protease active site in SVV 3C^pro^. The highly conserved phospholipid/RNA-binding motifs KFRDI and VGK are located on the face opposite from the active site in EV71 and many other picornaviral 3C^pro^.
(TIF)

S1 Table The cleavage junction sequences in the viral polyprotein and reported cleavage sequences from host proteins by SVV 3C^pro^. The cleavage sites down arrows are shown using down arrows and the numbers indicate the cleavage positions.
(DOCX)

S2 Table The list of SVV 3C^pro^ (wild-type and mutants) primers in this study. (DOCX)

S1 Dataset MS^2^ spectra of the untargeted lipidomics in this study. (XLSX)
