# Supplementary figures and images for: Allosteric regulation of Senecavirus A 3Cpro proteolytic activity by an endogenous phospholipid
Source: PLoS Pathog. 2023 May 30;19(5):e1011411. doi: 10.1371/journal.ppat.1011411 (PMC10256202; doi:10.1371/journal.ppat.1011411)

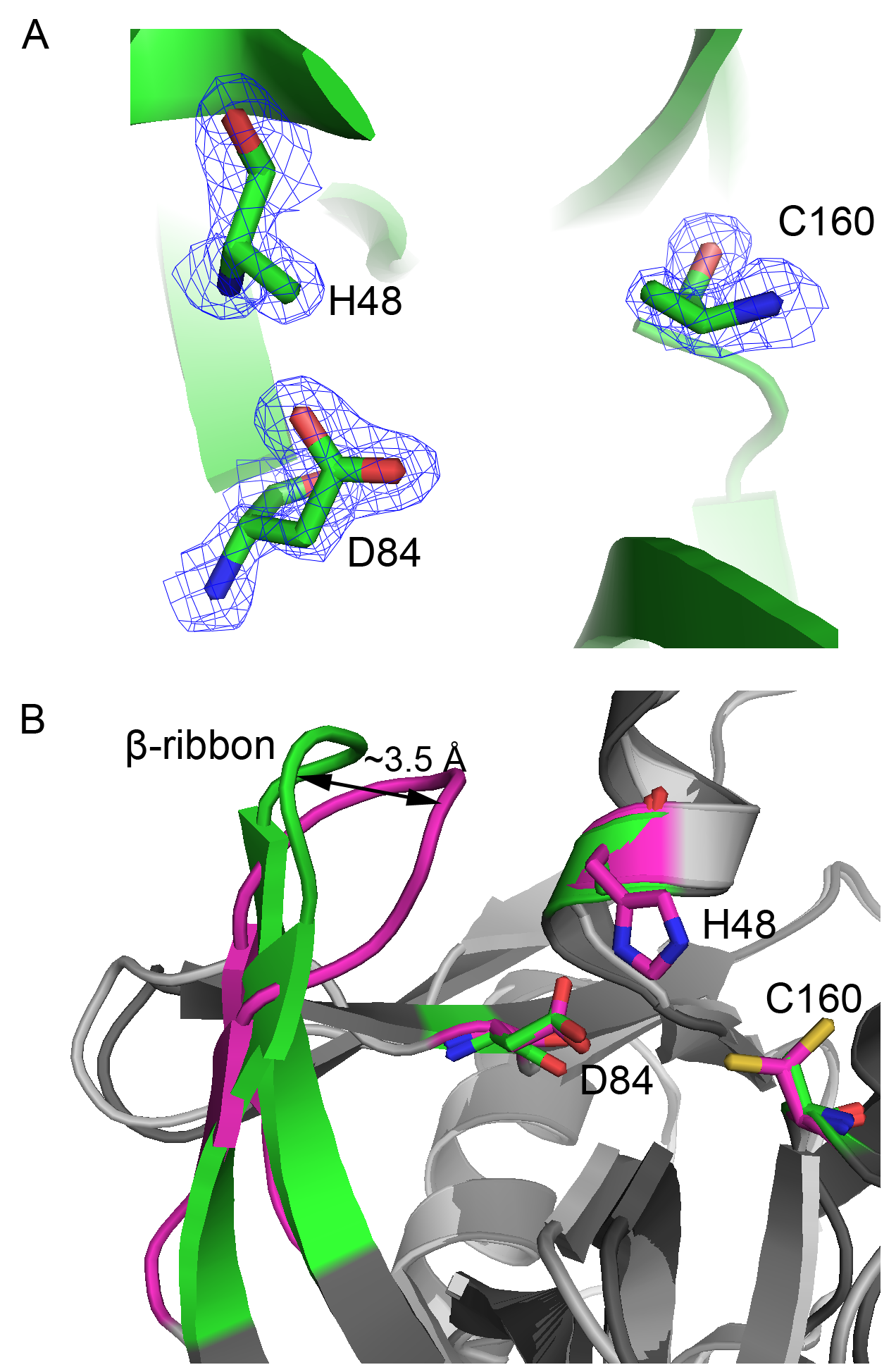

Supplement: S1 Fig — (A) Electron density map (2Fo-Fc) of the catalytic triad in PDB 6L0T, shown at a 1.5σlevel. It should be noted that Cys160 and His48 should be assigned as alanines judging from the electron densities. (B) Structural superposition of the active site between our SVV 3Cpro (light gray) and PDB 6L0T (dark gray). The catalytic triad and β-ribbon are highlighted in magenta and green, respectively. (TIF) [file ppat.1011411.s001.tif]

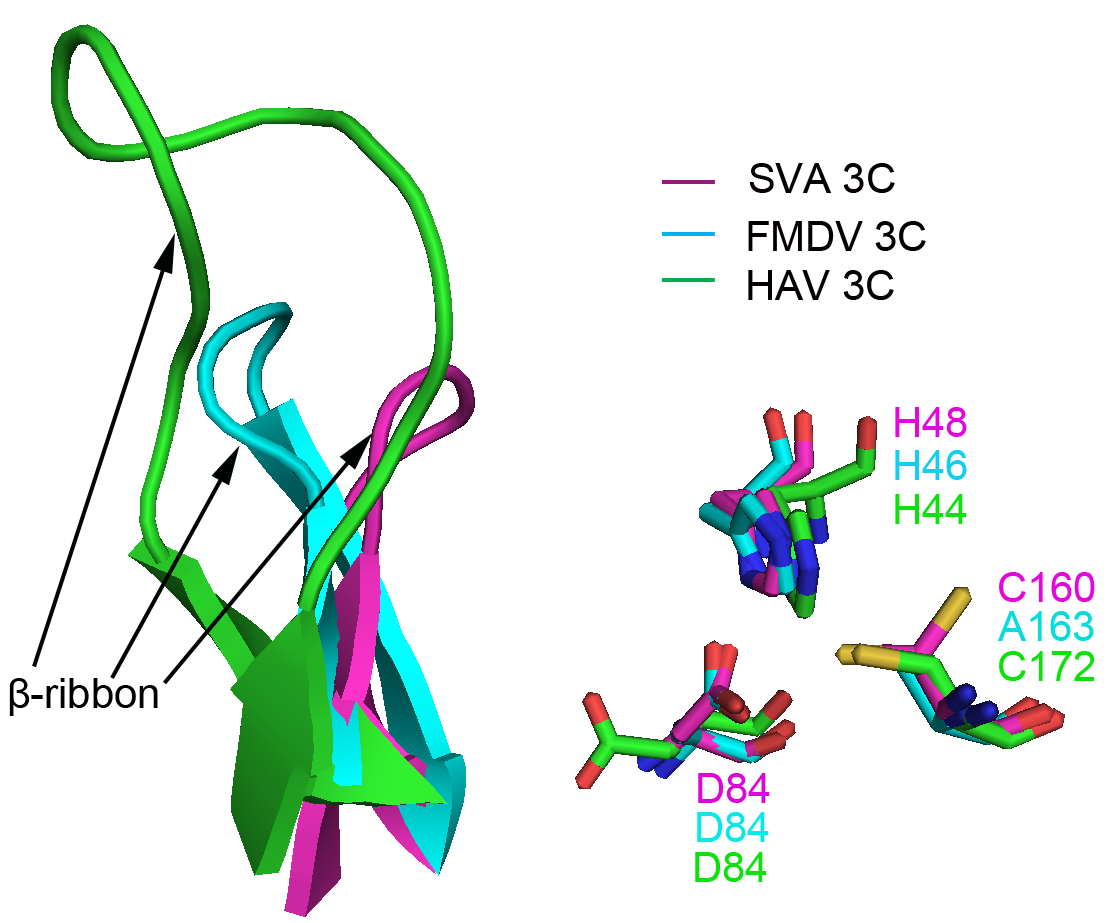

Supplement: S2 Fig — Structural superposition of the active site of SVV 3Cpro with those of representative homologs FMDV (PDB ID: 2J92) and HAV (PDB ID: 1QA7). (TIF) [file ppat.1011411.s002.tif]

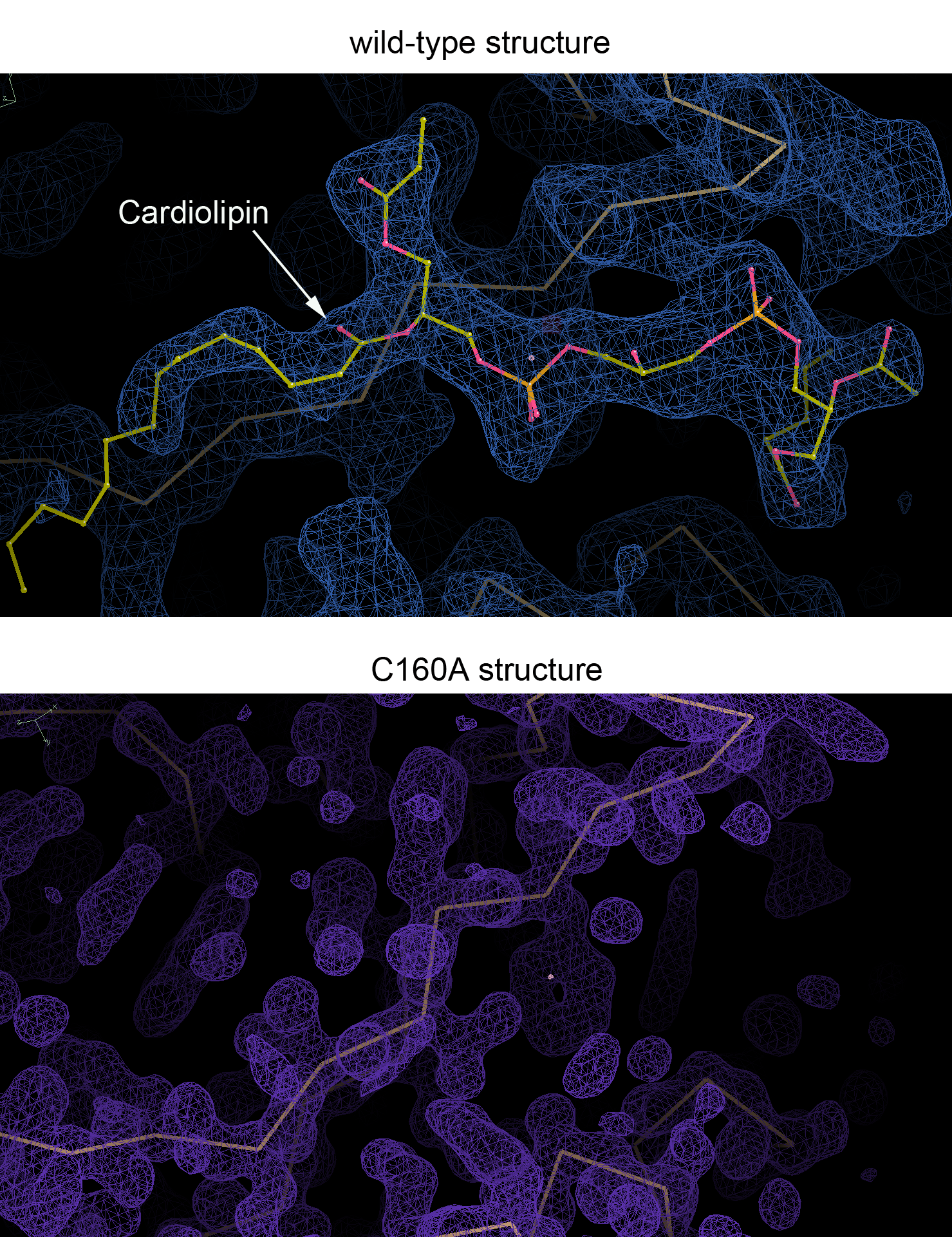

Supplement: S3 Fig — Electron density maps (2Fo-Fc) in both structures are shown at a 1.5σ level in Coot. (TIF) [file ppat.1011411.s003.tif]

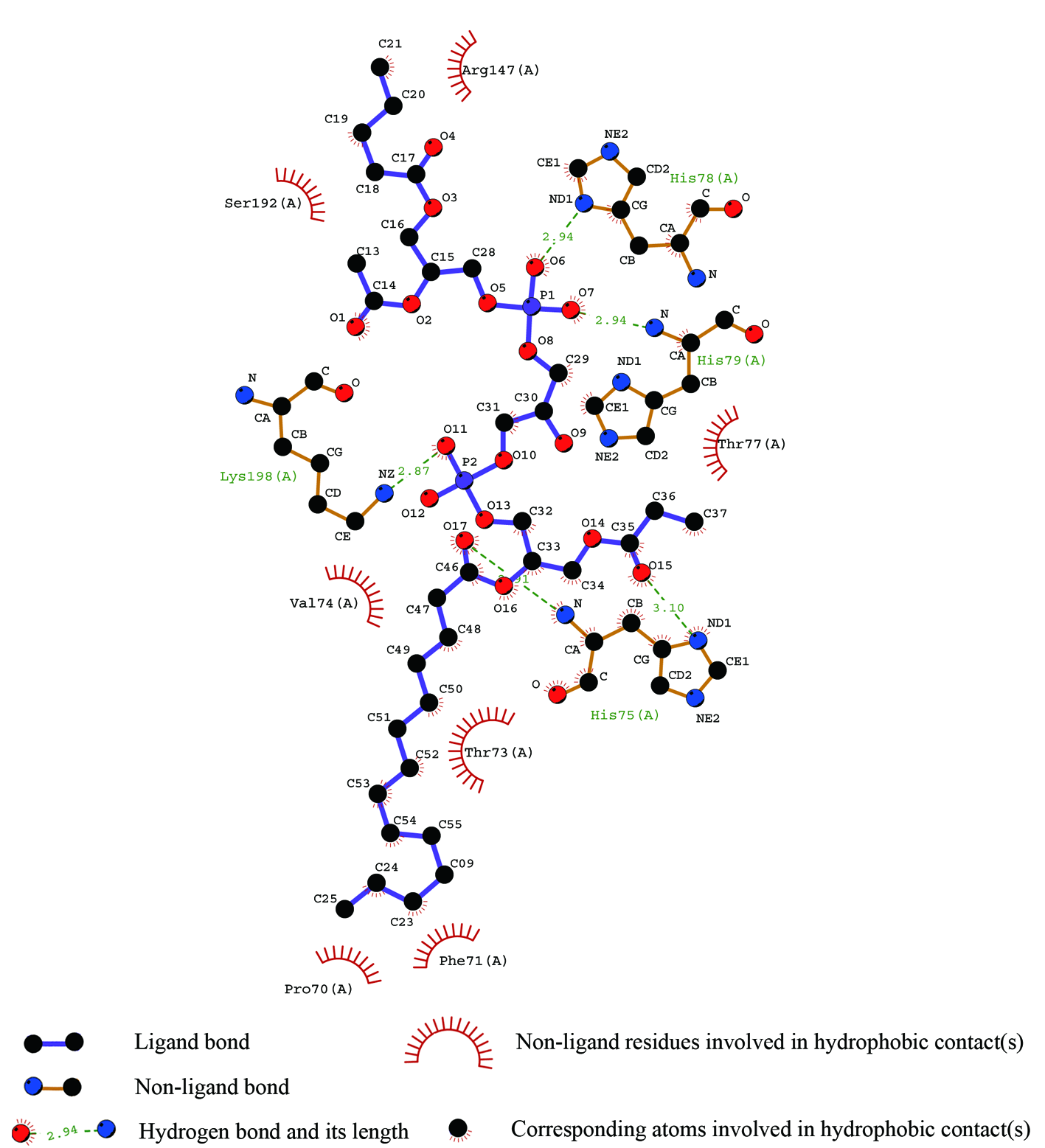

Supplement: S4 Fig — The H-bonds hydrogen-bonds (H-bonds) and hydrophobic contacts are calculated within 3.8 Å and 5.0 Å, respectively. (TIF) [file ppat.1011411.s004.tif]

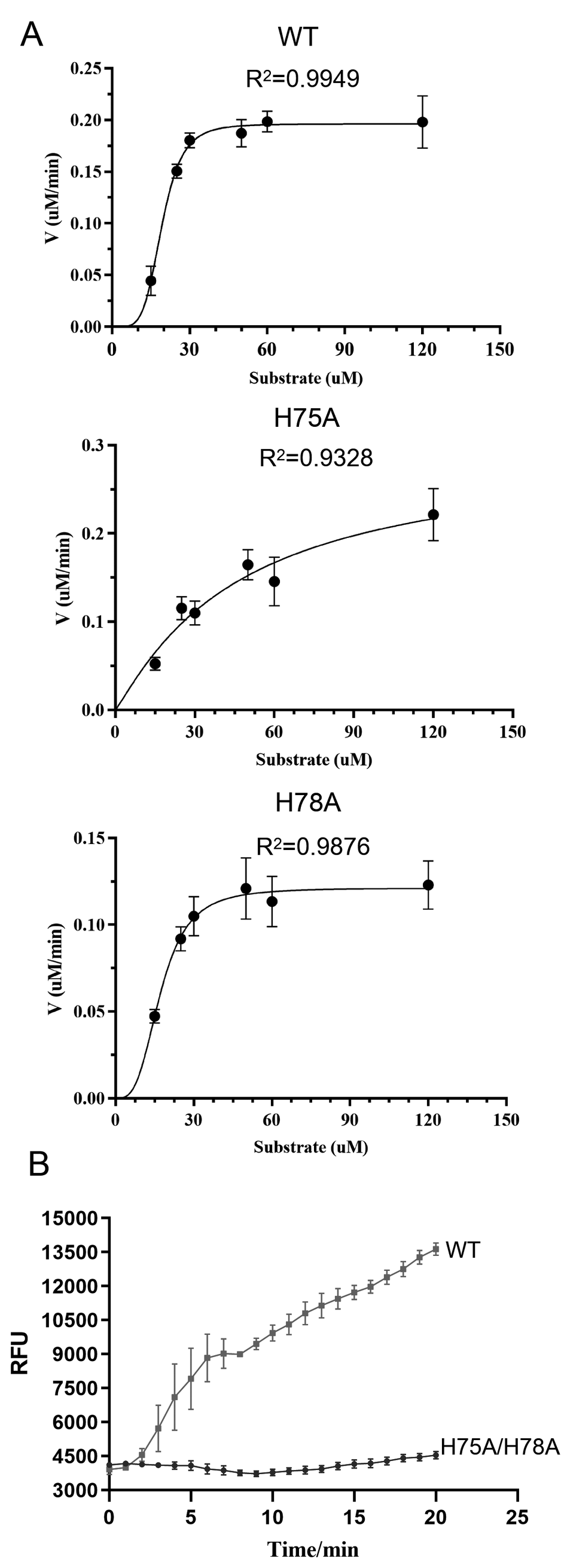

Supplement: S5 Fig — (A) The plots of initial rate (v) versus the substrate peptide concentration for wild-type (WT) and variants. See Materials and Methods for experimental details and Table 2 for the kcat/Khalf values determined for each variant. (B) Time course analysis of relative fluorescence unit (RFU) of the fluorogenic peptide cleaved by WT and H75A/H78A mutant. (TIF) [file ppat.1011411.s005.tif]

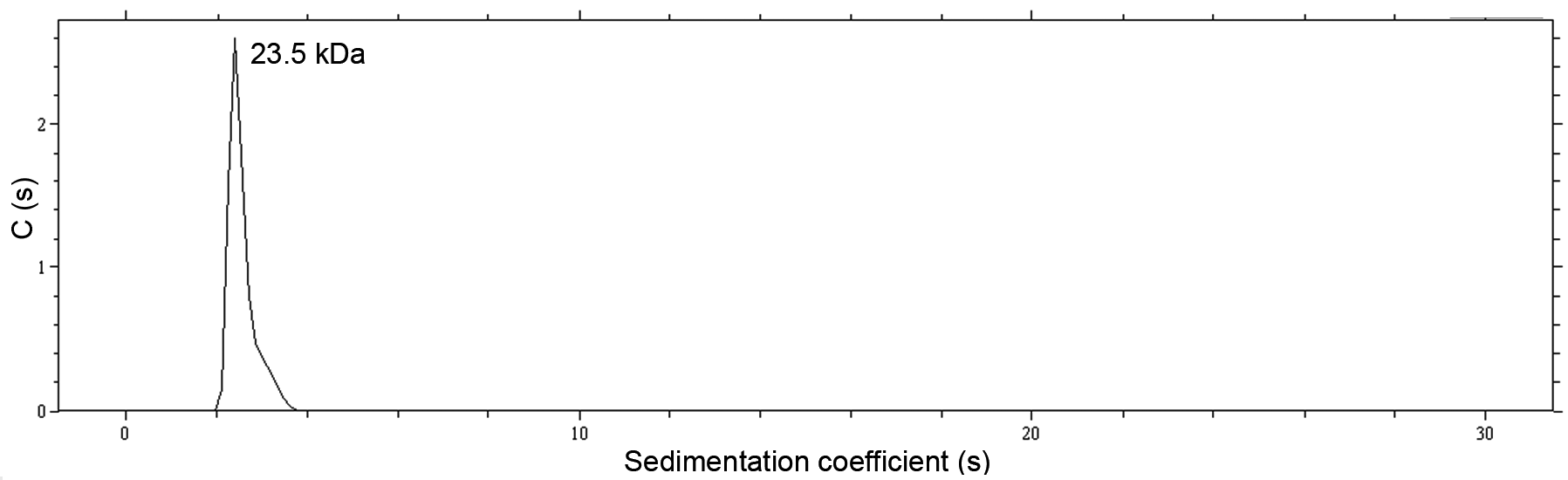

Supplement: S6 Fig — (TIF) [file ppat.1011411.s006.tif]

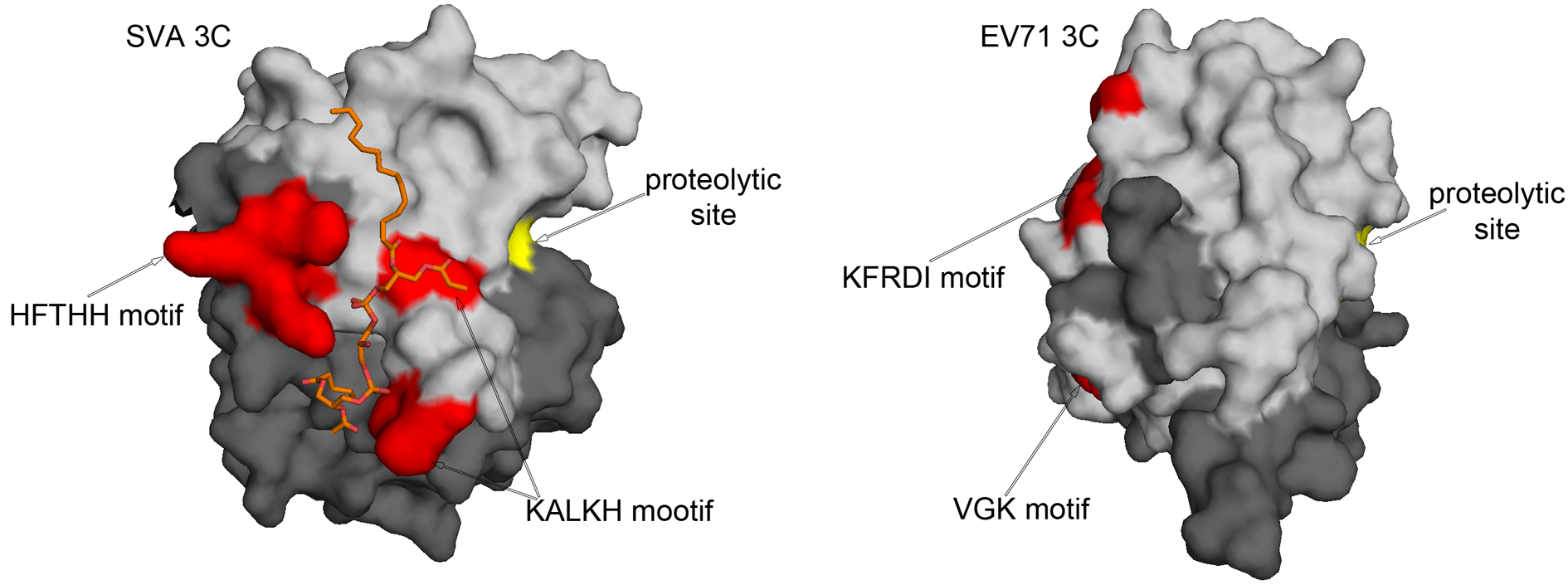

Supplement: S7 Fig — The phospholipid-binding motifs 75-HFTHH-79 and 198-KALKH-202 are located neighboring the protease active site in SVV 3Cpro. The highly conserved phospholipid/RNA-binding motifs KFRDI and VGK are located on the face opposite from the active site in EV71 and many other picornaviral 3Cpro. (TIF) [file ppat.1011411.s007.tif]
