# Supplementary material for: Allosteric regulation of Senecavirus A 3Cpro proteolytic activity by an endogenous phospholipid
Source: PLoS Pathog. 2023 May 30;19(5):e1011411. doi: 10.1371/journal.ppat.1011411 (PMC10256202; doi:10.1371/journal.ppat.1011411)
Supplement: S1 Table — The cleavage sites down arrows are shown using down arrows and the numbers indicate the cleavage positions. (DOCX) [file ppat.1011411.s008.docx]

**S1 Table.** The cleavage junction sequences in the viral polyprotein and reported cleavage sequences from host proteins by SVV 3C^pro^. The cleavage sites down arrows are shown using down arrows and the numbers indicate the cleavage positions.

| **Cleavage target** | **Cleavage sequence** |
| --- | --- |
| **Viral polyprotein junction** | |
| L-VP4 | DIVYELQ^79^↓GNVQTTS |
| VP2-VP3 | AGTDEEQ^434^↓GPIPTAP |
| VP1-2A | KQKMLMQ^937^↓SGDIETN |
| 2B-2C | RKLFKMQ^1074^↓GPMDKVK |
| 2C-3A | MQTLVLQ^1396^↓SPNENDD |
| 3B-3C | LSLMEMQ_1508_↓QPNVDMG |
| 3C-3D | EPLATMQ^1719^↓GLMTELE |
| **Host proteins** | |
| TANK | QEKFNME^272^↓FRDNPGN |
| TANK | ETLFEIQ^291^↓GIDPIAS |
| TRIF | GSIRTLQ^159^↓SNLGCLP |
| MAVS | MPVQETQ^148^↓APESPGE |
| GSDMD | PGAVSLQ^193^↓GQGQGHL |
| GSDMD | SEHLKFQ^277^↓SDGPAED |
| NLRP3 | MDGFDELQ^305^↓GAFDEHIG |
| PABPC1 | SPRWTAQ^437^↓GARPHPF |
| Nucleolin | GRAIRLELQ^545^↓GPRGSP |

L: Leader protein
