# Supplementary material for: Allosteric regulation of Senecavirus A 3Cpro proteolytic activity by an endogenous phospholipid
Source: PLoS Pathog. 2023 May 30;19(5):e1011411. doi: 10.1371/journal.ppat.1011411 (PMC10256202; doi:10.1371/journal.ppat.1011411)
Supplement: S2 Table — (DOCX) [file ppat.1011411.s009.docx]

**S2 Table.** The list of SVV 3C^pro^ (wild-type and mutants) primers in this study.

| **Primer** | **Sequence** |
| --- | --- |
| Wild-type Forward  Reverse | ACAGATTGGTGGATCCATGCAGCCCAACGTGG |
|  | TGGTGGTGCTCGAGTCATTGCATTGTGGCCAAAG |
| C160A Forward  Reverse | AAAGGATGGGCCGGCTCGGCC  GAGCCGGCCCATCCTTTGTAGGTCGTCAC |
| H75A Forward  Reverse | CCAAACGGTTGCCTTCACTCACCACG |
|  | GAGTGAAGGCAACCGTTTGGAAGGGCT |
| H78A Forward  Reverse | TCATTTCACTGCCCACGGTATTCCCACAG |
|  | GGAATACCGTGGGCAGTGAAATGAACCG |
| H79A Forward  Reverse | TCATTTCACTCACGCCGGTATTCCCACAGA |
|  | AATACCGGCGTGAGTGAAATGAACCGTTTGG |
| R147A Forward  Reverse | GAACTTACGCAGCCCTCTTTAGGTACAGGGTGAC |
|  | ACCTAAAGAGGGCTGCGTAAGTTCCTTGTTCAGA |
| K198A Forward  Reverse | GGACTAATCGCCGCCCTGAAACACCTCG |
|  | GAGGTGTTTCAGGGCGGCGATTAGTCCTAATTTTGAG |
| H202A Forward  Reverse | CTGAAAGCCCTCGGTGAACCTTTGGC |
|  | TTCACCGAGGGCTTTCAGGGCTTTGATTAGTC |
